# Fine-Tuning hIAPP Amyloidogenesis: Probing Molecular Mechanisms via Fluorinated Core Substitutions

**DOI:** 10.1021/acsbiomedchemau.6c00061

**Published:** 2026-05-05

**Authors:** Artem Pavlov, Hossein Batebi, Nicklas Österlund, Kevin Pagel, Boris Schade, Mario Schubert, Roland R. Netz, Beate Koksch

**Affiliations:** † Department of Chemistry and Biochemistry, 9166 Freie Universität Berlin, Berlin 14195, Germany; ‡ Department of Physics, Freie Universität Berlin, Berlin 14195, Germany; § Research Center of Electron Microscopy, Freie Universität Berlin, Berlin 14195, Germany

**Keywords:** amyloid formation, noncanonical amino acids, fluorine, aromatic interactions, IM-MS, NMR

## Abstract

Aggregation of human islet amyloid polypeptide (hIAPP, amylin) into amyloid fibrils is a hallmark of β-cell dysfunction in type 2 diabetes, yet the molecular determinants governing its aggregation pathways remain incompletely understood. Here, we investigate how systematic fluorination of Phe23—a key aromatic residue within the amyloidogenic core—modulates intra- and intermolecular interactions and thereby probes hIAPP self-assembly under physiologically relevant acidic and neutral pH conditions. Using a combination of Thioflavin T kinetics, scaling-exponent analysis, pyrene fluorescence, ion mobility–mass spectrometry, circular dichroism, ^19^F NMR spectroscopy, and molecular dynamics simulations, we show that fluorination of Phe23 reshapes aggregation behavior in a highly nonadditive manner. While wild-type and minimally fluorinated variants, at neutral pH, follow the surface-catalyzed secondary nucleation mechanism discussed well in the literature, higher degrees of fluorination progressively reduce monomer dependence and give rise to pronounced concentration-dependent, self-inhibiting aggregation behavior. Under acidic conditions, protonation of His18 leads to a divergent concentration dependence, with reduced monomer dependence for wild-type and minimally fluorinated peptides and enhanced concentration sensitivity for tetra- and penta-fluorinated variants. These concentration dependencies reflect differences in the conformational accessibility, flexibility, and oligomerization efficiency required for productive fibril formation under each pH condition. Together, these results identify Phe23 as a molecular switch that couples local interactions to global aggregation pathways and demonstrate how subtle chemical and environmental perturbations can modulate the productivity of hIAPP fibril formation.

## Graphical abstract

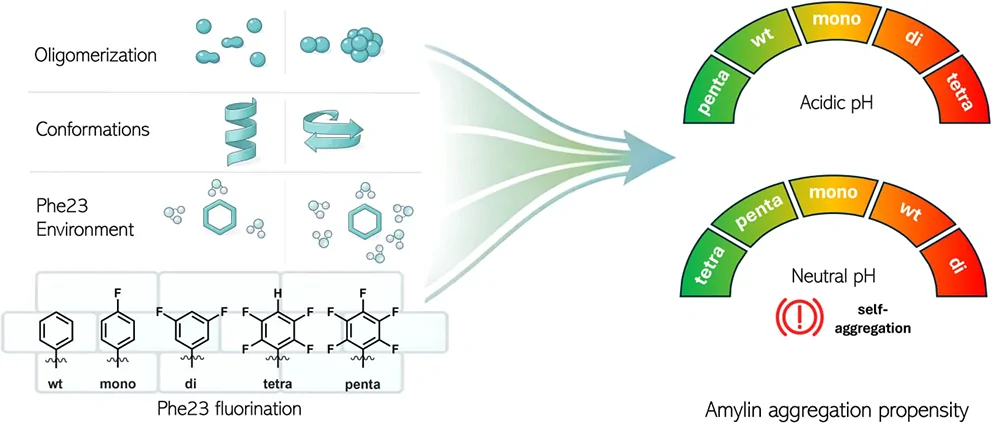

## Introduction

Amyloid formation is associated with a range of human diseases, most notably type 2 diabetes and Alzheimer’s disease. In both cases, the aggregation of intrinsically disordered peptides—human islet amyloid polypeptide (hIAPP, or amylin) and amyloid-β (Aβ), respectively—leads to the accumulation of fibrillar deposits that contribute to progressive cell dysfunction and death. In type 2 diabetes, hIAPP aggregation has been implicated in β-cell dysfunction and loss.^1–6^ Understanding the molecular mechanisms of amyloid formation is therefore of both fundamental and clinical importance, offering the potential to identify early aggregation intermediates as therapeutic targets and to guide the development of aggregation inhibitors.

Despite decades of research, the pathway of amyloid formation by hIAPP is incompletely understood. In vivo, amylin experiences distinct physicochemical environments, ranging from the acidic conditions ∼pH 5.8 of pancreatic β-cell secretory granules^7^ to the pH 7.4 of the extracellular space following secretion. The process involves a transition from a mostly disordered monomeric state through oligomeric intermediates to mature fibrils and is influenced by a combination of intra- and intermolecular interactions. Among these, hydrophobic and aromatic contacts are recognized as critical parameters but remain difficult to elucidate.

One segment of the hIAPP sequence that has received particular attention is the FGAIL motif, which constitutes the shortest identified amyloidogenic fragment and is largely responsible for the peptide’s self-assembly properties.^8,9^ Within and adjacent to this region lie several hydrophobic and aromatic residues thought to contribute to aggregation through noncovalent interactions (Figure 1). In particular, three aromatic residues in hIAPP—Phe15, Phe23, and Tyr37—have been proposed to engage in π–π stacking and other specific interactions during early aggregation stages.^9,10^ Systematic mutagenesis and binding studies have shown that among these, Phe23 plays a key role in hIAPP self-assembly, while its cooperative interaction with Phe15 is especially important in heteroassembly with Aβ40 and Aβ42. Although aromatic residues are not strictly required for mature fibril stability, their removal markedly alters the aggregation kinetics and morphology, underscoring their role in defining assembly pathways rather than end-state stability.^11^

**Figure 1:**
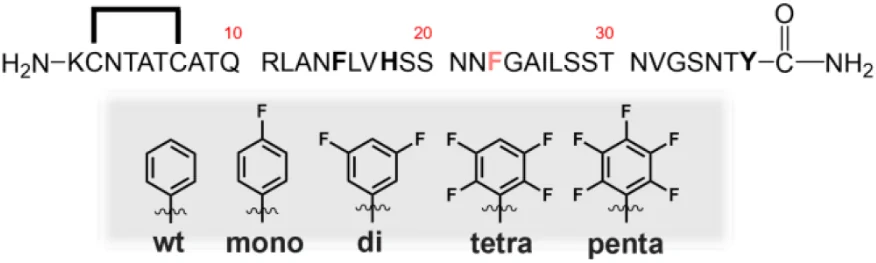
hIAPP sequence with Phe23 highlighted (top) and chemical structures of the fluorinated variants (bottom).

The interplay between these aromatic residues appears to be nonadditive in hIAPP. Previous findings suggest that they contribute to amyloid formation not simply through additive hydrophobic effects, but via cooperative mechanisms.^10^ Phe15, located in the helical N-terminal region, may affect early oligomerization;^9,12,13^ Phe23, centrally positioned within the FGAIL hydrophobic core, could modulate β-sheet formation and contribute to oligomerization as well;^9,14,15^ and Tyr37, at the C-terminus, may influence the organization of mature fibrils.^16^ Such nonadditive behavior points to conformational coupling between early and late aggregation events and highlights the need for residue-specific approaches capable of probing these subtleties in the full-length peptide.

Fluorine incorporation, in general, has been shown to influence multiple physicochemical properties of amino acids, including hydrophobicity, secondary structure propensity, and proteolytic stability, though the direction and magnitude of these effects are often context dependent.^17^ In the case of phenylalanine, fluorination alters the electrostatic potential of the aromatic ring, generating localized dipole moments and, in the case of the high degree of fluorination examples studied, reversing the intrinsic quadrupole moment of the aromatic system. This feature makes fluorinated analogues valuable tools for probing the role of aromatic residues in aggregation processes beyond what can be inferred from standard hydrophobic substitutions. Studies on short hIAPP fragments (e.g., hIAPP_20–29_ and hIAPP_22–29_) have shown that *para*-monofluorination and perfluorination of phenylalanine can accelerate aggregation, likely through increased hydrophobicity.^18,19^ Our previous work with the NFGAIL model and an extended library of fluorinated phenylalanines supported this view^20^ but also raised the question: whether such electronic modifications primarily affect intermolecular association patterns or intramolecular conformational preferences, and how these effects translate to the aggregation mechanism of the full-length peptide.

Here, we address this question using a combination of Thioflavin T kinetic analysis, pyrene fluorescence assay, ion mobility–mass spectroscopy, circular dichroism, and ^19^F NMR spectroscopy, as well as molecular dynamics simulations to probe how systematic fluorination at Phe23 alters the conformational preferences, oligomeric patterns, and assembly pathways of hIAPP. By comparing neutral and acidic pH conditions, we further show that protonation of His18, a key residue near the FGAIL motif, redefines the aggregation regime even for the wild type, providing an additional axis for modulating hIAPP self-assembly. Together, these experiments reveal that Phe23 functions as a molecular switch that governs cooperativity in the fibrilization process between intrachain conformational preferences and interchain association, thereby determining whether hIAPP proceeds along its canonical fibrilizaation pathway or diverges into self-inhibitory, off-pathway assemblies.

## Results

### Macroscopic Analysis of Aggregation Kinetics

To evaluate the macroscopic effect of fluorine substitution in the Phe23 ring on hIAPP aggregation, we first performed ThT kinetic assays. Experiments were conducted in 30 mM sodium acetate buffer (pH 5.3) and 10 mM phosphate buffer (pH 7.4) to approximate the acidic environment of β-cell secretory granules and the near-neutral extracellular milieu, respectively. The peptide concentration ranges of 15–150 μM at pH 5.3 and 2.5–150 μM at pH 7.4 were chosen in line with prior in vitro studies of amylin aggregation,^21,22^ where micromolar concentrations enable reproducible kinetic analysis, while higher concentrations were included to probe potential changes in aggregation behavior arising from enhanced intermolecular interactions. At neutral pH, hIAPP aggregates rapidly even at low micromolar concentrations, allowing reliable kinetic analysis down to 2.5 μM. In contrast, under acidic conditions, protonation of His18 increases electrostatic repulsion between monomers and raises the effective nucleation barrier;^23–25^ in other words, no aggregation was observed for concentrations below 15 μM for several days.

Under acidic conditions, increasing fluorination produces a clear acceleration of aggregation up to the tetra-fluorinated (tetra) variant, whereas the penta-fluorinated (penta) peptide deviates from this trend and aggregates at a rate comparable to the wild type (Figure 2A). Notably, the penta variant is the only peptide under these conditions that displays anomalous concentration dependence.

**Figure 2:**
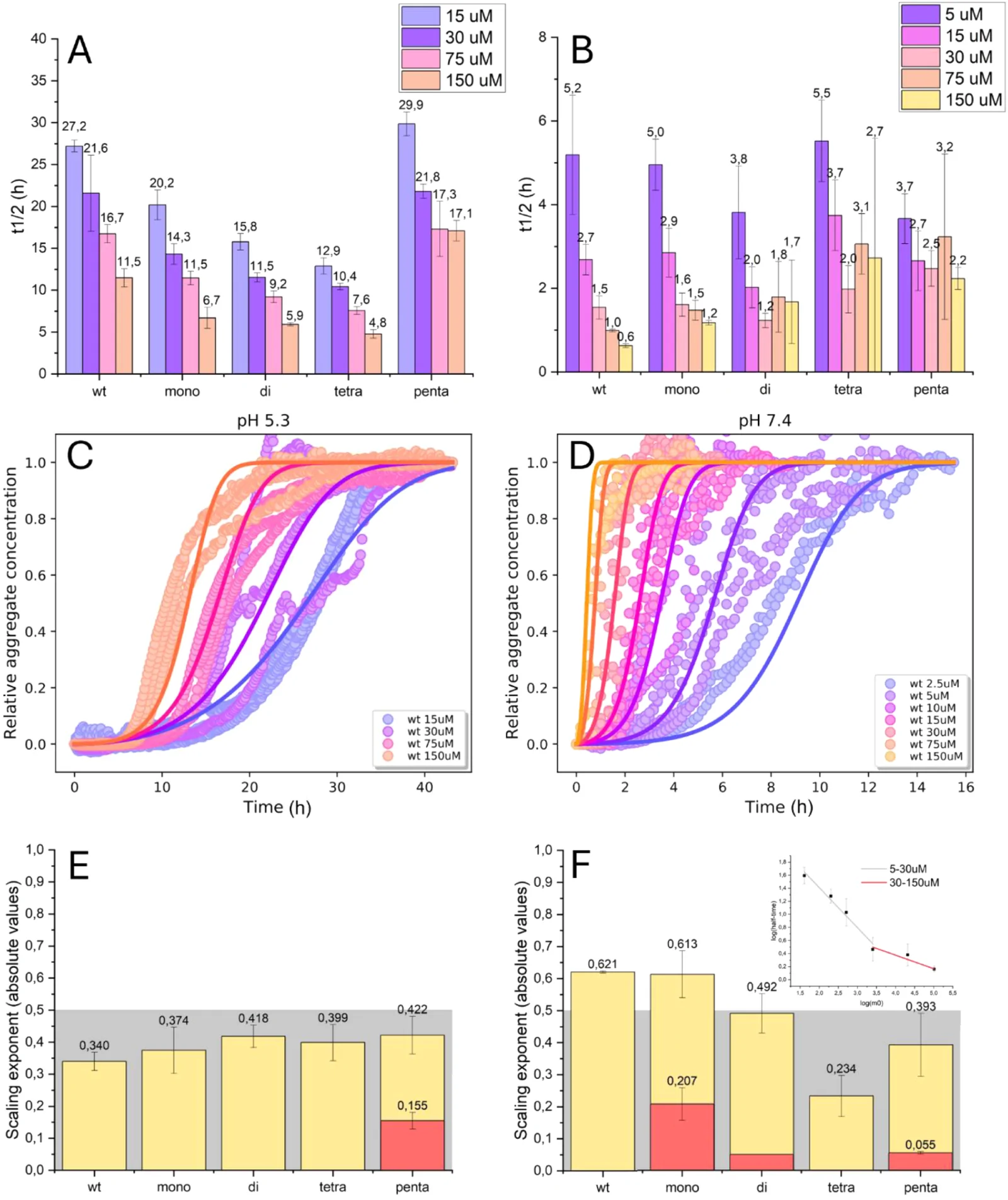
Macro- and microscopic analyses of aggregation kinetics of hIAPP variants at 37 °C at different pH values: 5.3 (A,C,E) and 7.4 (B,D,F). Half-time dependence of amyloid formation on the concentration of hIAPP variants at pH values of 5.3 (A) and 7.4 (B). Normalized ThT fluorescence intensity versus time for wt peptide at pH values of 5.3 (C) and 7.4 (D); 3–6 replicates were carried out. Absolute scaling exponent values versus hIAPP variants at a pH of 5.3 (E) and 7.4 (F); effect of high concentration depicted by red columns; an example of the slope change of the log–log plot of *t*_1/2_ versus *m*_0_ for the mono variant (inset F).

In contrast, at neutral pH (7.4), no clear monotonic trend is observed (Figure 2B): in the 5–30 μM interval, the mono-fluorinated (mono) variant aggregates at a rate similar to the wild type, the di-fluorinated (di) peptide aggregates faster, the tetra peptide aggregates more slowly, and the penta variant, independent of initial concentration, displays similar *t*_1/2_values. These results indicate that previously established fluorination trends for short model peptides (e.g., NFGAIL, NFGAILSS) do not apply to full-length hIAPP.^18–20^

### Microscopic Mechanism from Kinetic-Model Fitting

To relate the macroscopic aggregation behavior to underlying microscopic steps, we analyzed the ThT traces using the AmyloFit^26^ platform, a fitting that used a model including primary nucleation, multistep secondary nucleation, and elongation. This assigns wt hIAPP to a surface-catalyzed secondary nucleation mechanism, consistent with earlier reports for hIAPP^21,22^ as well as for Aβ peptides.^27,28^ The aggregation of wt hIAPP has a good global fit across the entire tested concentration range and at both pH conditions, by a model dominated by multistep secondary nucleation (Figure 2 C and D). For the fluorinated variants, however, this mechanistic model holds only in certain cases. The mono- and the di-peptides can be satisfactorily fitted at pH 5.3 across all tested concentrations and at pH 7.4 only within the 5–30 μM interval.

In contrast, the tetra- and penta-variants cannot be adequately captured by this model under both pH regimes; at neutral pH, the fitting with this model fails completely (Figure S1). This failure to be fit strongly suggests that high degrees of Phe23 fluorination alter key microscopic steps of the aggregation process.

### Microscopic Mechanism from Scaling-Exponent Analysis

To further probe these deviations, we calculated the scaling exponent γ, a parameter that describes the dependence of the characteristic aggregation time on the initial monomer concentration via the relation $t_{1/2}∼m_{0}^{\gamma }$ . The exponent, obtained from the slope of the log–log plot of *t*_1/2_ versus *m*_0_, reflects the effective monomer reaction order of the dominant nucleation pathway and therefore serves as a sensitive indicator of the underlying microscopic aggregation mechanism.^29–31^

For wt hIAPP at pH 7.4, *γ* = −0.621 (Figure 2F), which lies within the expected range (−0.5 to −1.5) associated with surface-catalyzed secondary nucleation.^21,22^ While the scaling exponent may vary across different concentration intervals for some aggregating systems, the wt peptide exhibits a constant slope across the entire tested range (2.5–150 μM; Figure S2). Deviations from such linear behavior typically appear as a discontinuity of the slope or flattening of the log–log plot and reflect shifts in the dominant microscopic aggregation steps: steeper slopes at low concentrations indicate strong monomer dependence associated with unsaturated secondary nucleation (∼−1.5), whereas flattening at higher concentrations indicates reduced monomer dependence, often arising from saturation of surface-mediated processes or increased fragmentation (∼−0.5).^27,28^ In contrast to wt, all fluorinated analogues at pH 7.4 progressively diverge from this canonical regime showing the flattening at higher concentrations (red columns) observed for mono, di, and penta (Figure 2F). It should also be pointed out that tetra displays a remarkably small slope of the log–log plot that corresponds to decreased concentration dependency (Figure S2).

At pH 5.3, the behavior differs: all peptides, including wt, exhibit scaling exponents less negative than −0.5, indicating reduced monomer dependency compared to neutral pH (Figure 2E). Interestingly, the values of the slopes remain relatively uniform across variants, except for the penta, which at higher concentrations shows a scaling exponent approximately 2-fold lower than all of the others under this pH regime.

The comparison between the pH conditions highlights a fundamental divergence. Under acidic pH conditions, it can be broadly stated that wt, mono, di, and tetra behave in a similar manner, whereas penta displays different characteristics. Under neutral pH conditions, wt shows distinct behavior from all fluorinated variants.

### Rationale behind Inhibition Model

Taken together, the nearly identical *t*_1/2_ values at the highest peptide concentrations (Figure 2 A and B), the pronounced flattening of the *t*_1/2_ versus *m*_0_ dependence (Figures 2 E and F; S2), and the inability of classical secondary-nucleation models to capture these features (Figure S1), all point to a scenario in which increasing peptide concentration progressively limits its own aggregation namely, in the case of the penta variant under acidic conditions and all fluorinated variants under neutral conditions. To represent such apparent self-inhibition within the available kinetic frameworks, we therefore evaluated a model within the Amylofit^26^ platform that includes an inhibition term. This framework allows for the presence of species whose concentration increases with total peptide and that diminish the effective rate of productive fibril formation, providing a straightforward way to capture the apparent self-inhibition suggested by the macroscopic observations. Applying this model restored satisfactory global fits and yielded physically interpretable parameters, indicating that elongation and/or secondary nucleation are the primary steps subject to inhibition (Figure S1). The inhibition-containing model demonstrates that the degree of inhibition converged upon over the course of fitting is minimal for the mono-fluorinated variant and progressively more pronounced for di-, tetra-, and penta-fluorinated analogues. Notably, this apparent self-limiting behavior is not observed for the wild-type peptide at comparable concentrations, arguing against a purely diffusion-limited or universally concentration-driven effect. Instead, the data are more consistent with a fluorination-dependent alteration in the geometry and pattern of intermolecular interactions at the fibril surface. Increasing the fluorination at Phe23 modifies the electrostatic potential and steric profile of the aromatic ring, which may affect how monomers engage with catalytic fibril interfaces during secondary nucleation under both pH regimes. If surface binding becomes more persistent yet less productively oriented, surface sites may effectively become saturated by nonproductive assemblies, reducing the efficiency of monomer conversion into growing fibrils. In this framework, the inhibition term captures a sequence-specific perturbation of surface-catalyzed secondary nucleation rather than a generic concentration-induced limitation.

### Hydrophobic-Pocket Formation Detected by Pyrene Fluorescence

Pyrene fluorescence was used to probe the formation of hydrophobic microenvironments, with particular attention to the possibility of peptide-induced micelle formation, as previously reported.^32–34^ Pyrene is a polarity-sensitive fluorescent probe, and its vibronic emission intensity ratio (I_1_/I_3_) decreases as the local environment becomes less polar, with micelle formation typically producing a pronounced reduction relative to the aqueous value.^33,34^ Pyrene was titrated with different concentrations of peptide at neutral pH. The I_1_/I_3_ ratio remained near the aqueous value (≈ 1.8) for all variants in the range of 1–30 μM, except penta, which decreased to ≈ 1.5 at 30 μM (Figure S3). While this decrease indicates the emergence of a moderately hydrophobic environment, despite earlier reports, the magnitude of the change is inconsistent with micelle formation, which typically produces substantially lower I_1_/I_3_ values (≤1.3). No change occurred for any sample at pH 5.3. These results indicate that fluorination does not promote classical micelle formation but instead produces modest hydrophobic clustering, consistent with the formation of early oligomeric assemblies rather than detergent-like aggregates for both pH regimes.

### Effects on Oligomerization as Monitored by Native Mass Spectrometry

To explore the underlying aspects of the observed aggregation kinetics, specifically the pattern of early oligomerization, we employed native ion mobility mass spectrometry (IM–MS). IM–MS has previously been used to detect oligomer formation in amyloidogenic peptides^35,36^ including IAPP.^37,38^ Under the present conditions (15 μM freshly prepared peptide, 200 mM ammonium acetate pH 7.4), wild-type IAPP was detected primarily as a monomer, with smaller amounts of dimers also observed (Figure 3).

**Figure 3:**
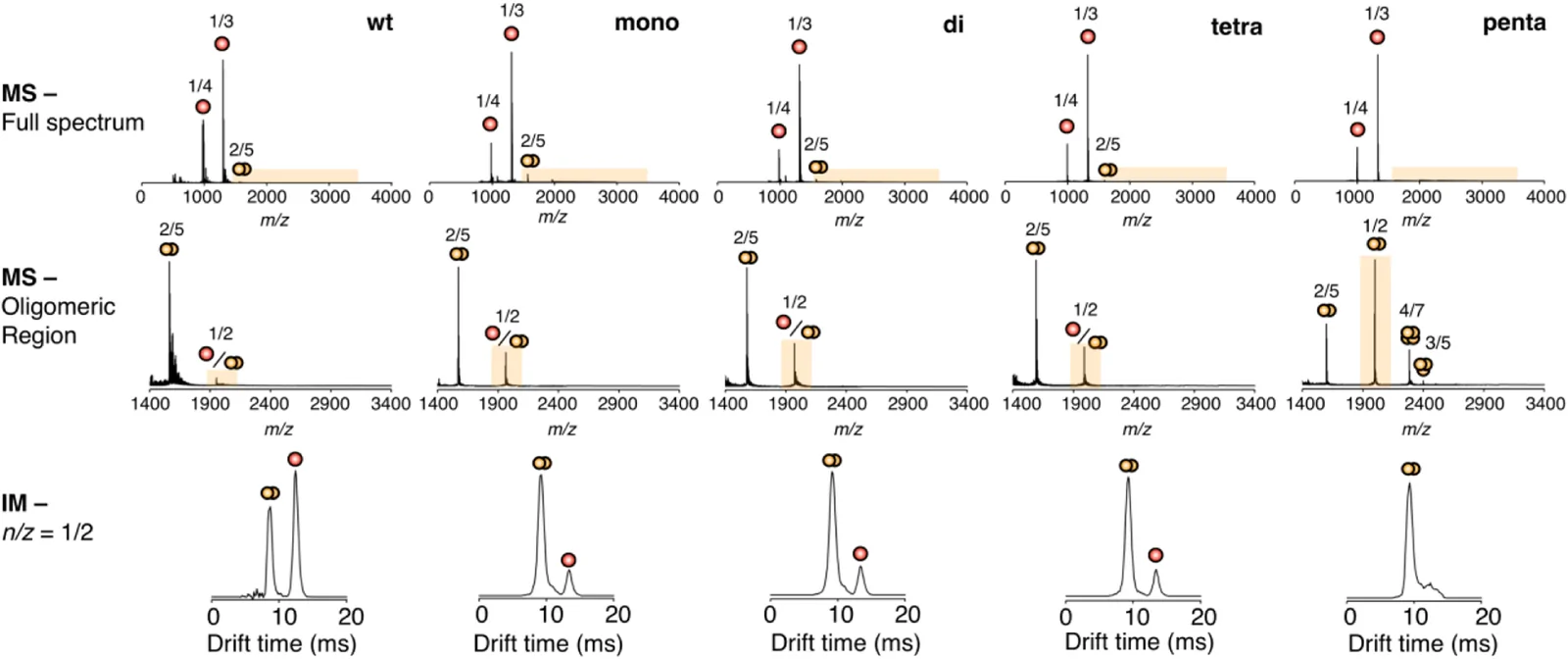
Native IM-MS of wild type and fluorinated variants, all in 200 mM ammonium acetate, pH 7. For each variant, the full mass spectra are shown (top), the oligomeric region (center), and the mobilogram for the n/z = 1/2 charge state (bottom).

Dimerization increased slightly for the mono, di, and tetra variants under the same sample conditions (Figure 3, bottom). In contrast, the penta-fluorinated variant exhibited a distinct shift in its oligomeric distribution toward the tetrameric form of the peptide. This shift toward tetrameric species is consistent with the altered hydrophobic environment detected by pyrene fluorescence and correlates with the reduced monomer dependence observed in kinetic scaling analysis. The preferential population of defined oligomeric states may limit the pool of monomers available for productive elongation or secondary nucleation, thereby contributing to the apparent self-limiting aggregation behavior at neutral pH (Figure 2, part B). The oligomeric distribution for the wild-type peptide remained unchanged when acidifying the sample solution to pH 5. In contrast, the higher charge states observed in the penta variant were completely depopulated upon acidification (Figure S5), leading to a distribution similar to the wild type. These results support the conclusion that self-limiting oligomers form at pH 7.4 for all fluorinated variants.

### Secondary-Structure Evolution from Circular Dichroism (CD)

We followed the conformational impact of Phe23 substitution on the initial phase of IAPP amyloid formation and its end point equilibrium by circular dichroism measurements. Initial secondary structures were determined immediately after resuspension in buffers of pH 5.3 and 7.4, with peptide concentrations of 30 μM. Samples were incubated in the cuvette within the CD device thermostat at 37 °C until no changes occurred (pH 5.3 samples were incubated in the thermostatic cabinet). The 150 μM samples were also measured, intended to correspond to the high-concentration condition (Figures S6-S7).

Initial conformation at pH 5.3 and a concentration 30 μM (Figure 4A) provides spectra characterized by a minimum near 200 nm and a shoulder around 220 nm for all variants, features commonly associated with disordered conformations containing residual helical contributions.^39,40^ At pH 7.4, the overall spectral shape remains similar, although di-, tetra-, and penta variants display reduced intensity around 200 nm, suggesting altered conformational distributions (Figure 4B). The reduced spectral intensity may reflect redistribution between monomeric and oligomeric states, consistent with the altered oligomer populations observed by IM–MS, although CD alone does not allow direct quantification of oligomer participation.

**Figure 4:**
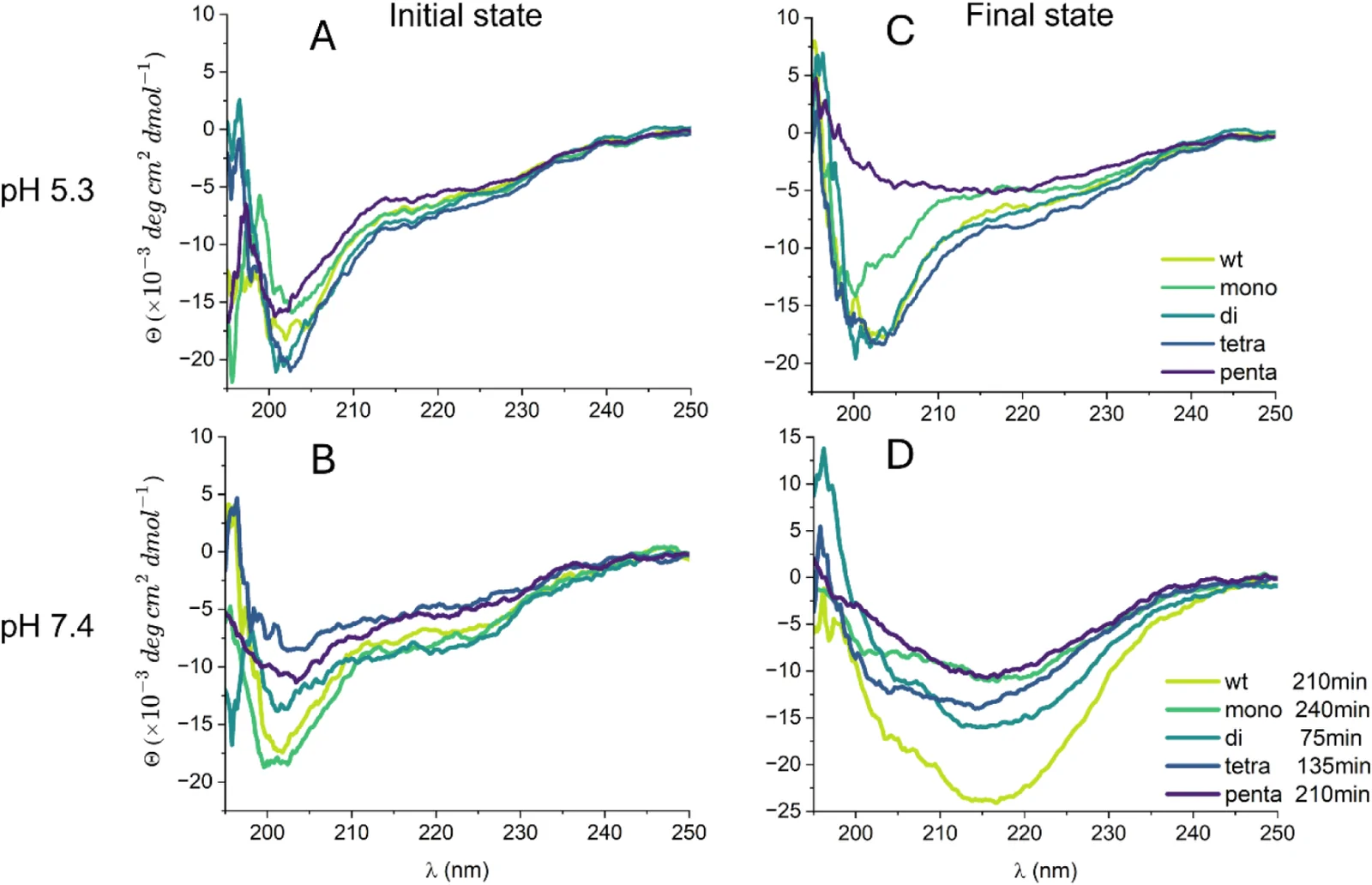
Circular dichroism spectra of hIAPP. Spectra (A, B) were measured immediately after preparation; spectra (C, D) show final states after incubation at 37 °C (for C – 4 days, for D – times in minutes are indicated in parentheses). Thirty μM in acetate buffer, pH 5.3 (A, C), and in phosphate buffer, pH 7.4 (B, D).

Surprisingly, at pH 5.3, all spectra except that of the penta variant indicate a random-coil conformation that persists even after 4 days of incubation at 37 °C (Figure 4D), while ThT indicates completed fibrilization for these conditions at this time point (Figure 2A). The only exception was the penta hIAPP analogue, which lost the 200 nm minimum and developed a positive β-sheet maximum around 195 nm and a negative flattened minimum near 215 nm. Upon incubation at room temperature for 1 week, on the other hand, similar qualitative behavior was observed except that the tetra variant developed β-sheet character (Figure S6A). Essentially, only increased concentration made the β-structures observable for pH 5.3 for all hIAPP analogues (Figure S6B). At neutral pH, all peptides ultimately adopted β-conformation to some extent, with a minimum at 215 nm. However, all variants except penta have an inclusion of a minimum at 205 nm that might come from α-helices. The order of the speed of transformation is as follows: di < tetra < wt ≈ penta < mono (Figure 4F).

As expected, the increased concentration mostly induced an early β-sheet structure appearance. Thus, at pH 5.3, the β-sheet signature was observed for the first time for all variants at 150 μM. Interestingly, the shape of the spectra was uniform among the variants, and the final conformation was adopted instantly. The di variant served as an exception, displaying an α-helix conformation that was unusually stable and lasted as the main conformational fraction for the time scale of *t*_1/2_ of aggregation (Figure S6C). At neutral pH, a similar pattern was observed, with almost instant adoption of a uniform classical β-sheet as a final state among the variants; conversely, some initial states showed a broad distribution of conformations (Figure S7).

### Local Environment of Phe23 from ^19^F NMR

^19^F NMR spectra were measured to carry out chemical-shift comparison of native (sodium acetate buffer, 30 mM, pH 5.3) and denatured (6 M Gdn·HCl) states of fluorinated hIAPP analogues, which makes possible the analysis of the local structure. The data show that whereas the mono- and the di-hIAPPs were virtually unaffected by denaturation (less than 0.1 ppm difference) (Figure 5A and B), tetra- and penta-fluorinated variants exhibit a notable upfield shift (∼0.5 ppm) for the denatured state (Figure 5C and D). The latter indicates a distinct change upon unfolding in the magnetic shielding environment of the highly fluorinated phenyl ring, consistent with its distinguished environment in the native state described by simulations (see below). Based on the combined data of our experiments, we assume that mono- and di variants behave in a similar manner to wt hIAPP, while tetra and penta acquire unique traits that may be caused by alterations in the local structure of the Phe23 region.

**Figure 5:**
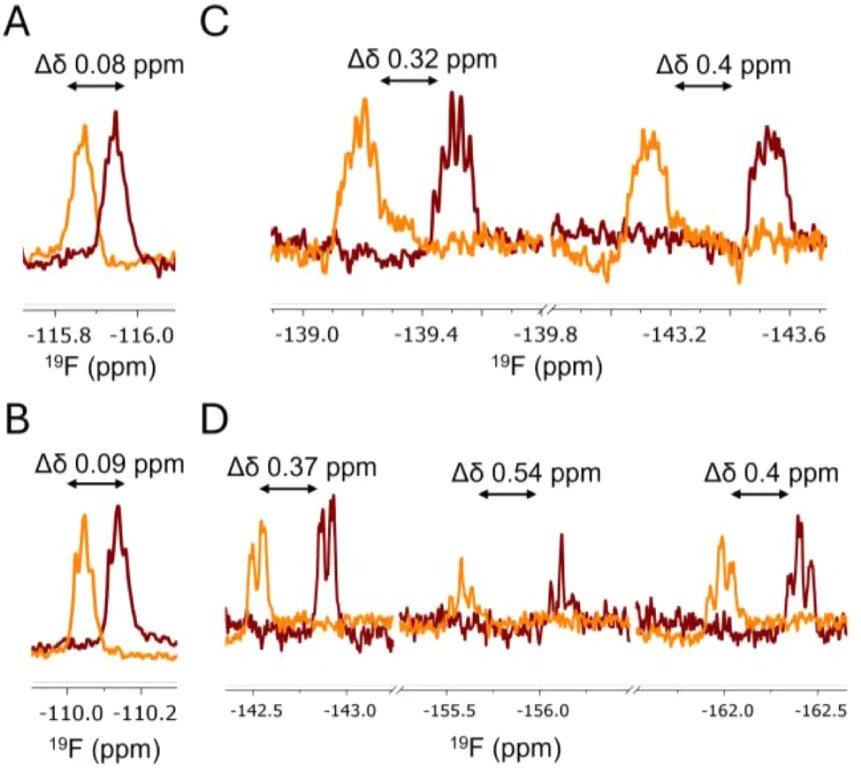
Conformational screening of hIAPP variants at the Phe23 region by ^19^F NMR: red – pH 5.3 acetate buffer 30 mM (measured within the lag time), orange – Gdn*HCl 6M; A – mono, B – di, C – tetra, D – penta.

### Structural Insights from Molecular-Dynamics Simulations

Molecular-dynamics simulations were performed to probe the effect of Phe23 fluorination on local structure and self-assembly (Figure 6; simulations were conducted for wt, hIAPP with tetra- and penta-fluorinated Phe23; mono- and difluorinated variants were not simulated).

**Figure 6:**
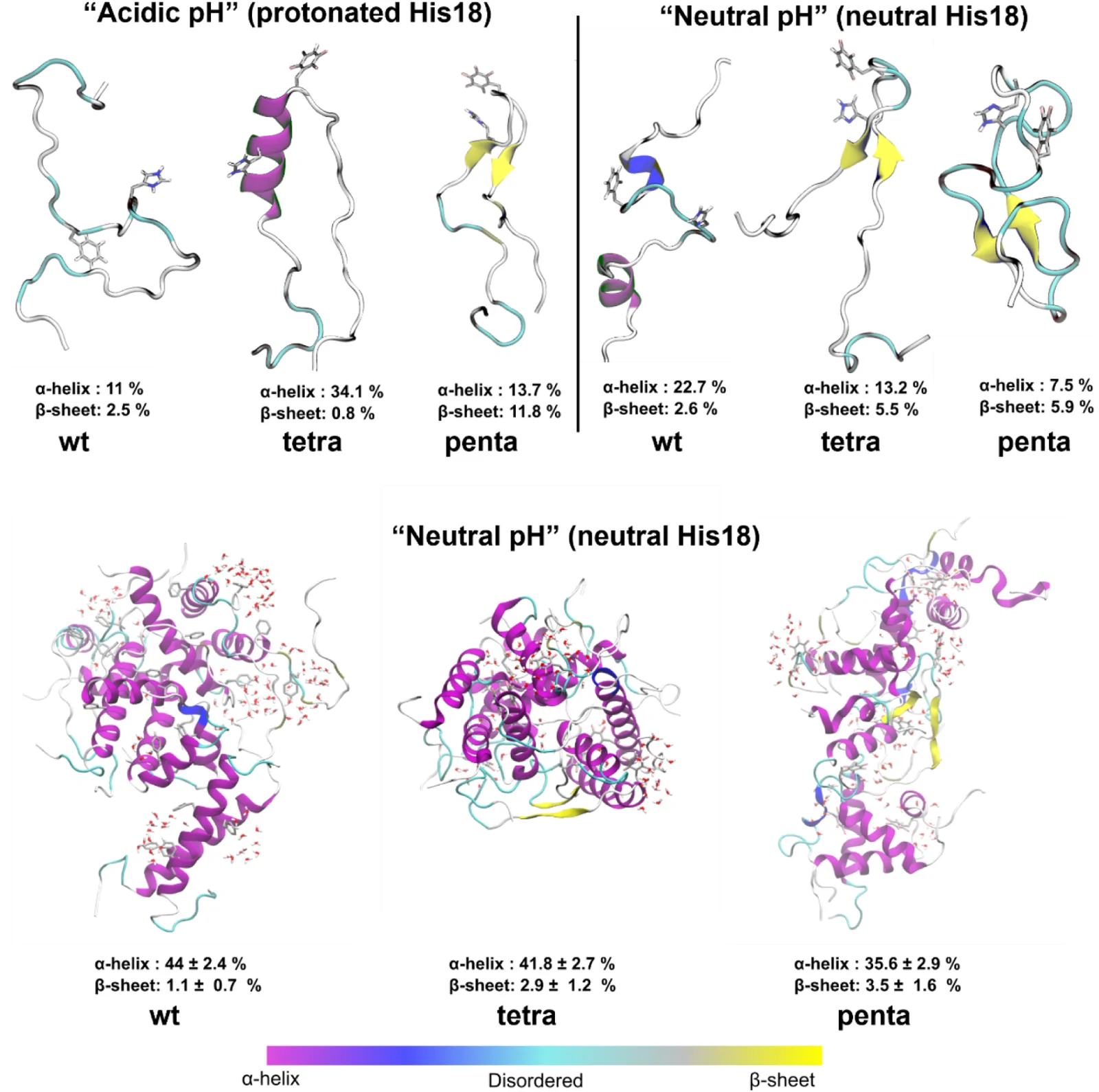
Representative conformations of a single molecule of hIAPP peptide (top) and assembly behavior of 10 hIAPP chains (bottom) from 1 μs molecular-dynamics simulations for wt, tetra, and penta.

The single-molecule peptide simulations indicate that for “acidic conditions” (protonated His18), tetra-fluorinated Phe23 increases local helical propensity in the ∼15–25 region, whereas penta-fluorinated Phe23 promotes β-strand propensity in the FGAIL segment (Figure 6, top left). In contrast, for “neutral pH” (neutral His18), decreased helical and increased β-strand propensities are observed for both tetra- and penta variants (Figure 6, top right). These findings provide an explanation of the difference in the experimental tetra and penta hIAPPs ThT kinetics behavior observed for different pH regimes.

In our multimeric simulations performed at neutral pH, the same shift in propensities occurred for the fluorinated variants. Also, both variants show a notable increase in solvent exposure of the Phe23 side chain compared to the wild type based on the computed solvent-accessible surface area (SASA) values and broader distributions of water coordination around this residue (Figures S8-S11), which may be the reason for the enhanced intermolecular interaction. The tetra variant forms compact assemblies, whereas the penta system produces larger, more heterogeneous aggregates that remain partially hydrated and extended (Figure 6, bottom). These variant-specific differences align closely with pyrene experimental data that showed a decreased polarity of the environment introduced by the penta sample, confirming that the perfluorination of Phe23 prevents efficient burial of the hydrophobic core in tight assemblies and promotes solvent-accessible, β-prone conformations. Our simulations of single-peptide and multimeric models demonstrate that fluorination at Phe23 modulates the conformational preferences of tetra- and pentavariants in the same manner at neutral pH, while revealing divergent effects between them at acidic pH.

### Cryogenic TEM

This technique was employed to confirm the presence of amyloid fibrils as a final state of aggregation and to assess the morphological features of the fibrils formed by wt hIAPP and its fluorinated Phe23 variants. No striking differences in overall morphology were observed, although all samples exhibited specific fibrillar structures (Figure 7). However, subtle trends were noted: wild-type fibrils appeared shorter and exhibited a higher degree of lateral association, suggesting a stronger tendency to form bundled aggregates (Figure 7A-C). In contrast, fibrils formed by the fluorinated variants appeared longer and more flexible. It is important to note that, although these observations are quite subtle, they are consistent across a wide range of observations and therefore likely reflect underlying differences in the electrostatic potential and intermolecular binding characteristics of the phenylalanine side chain altered by fluorination.

**Figure 7:**
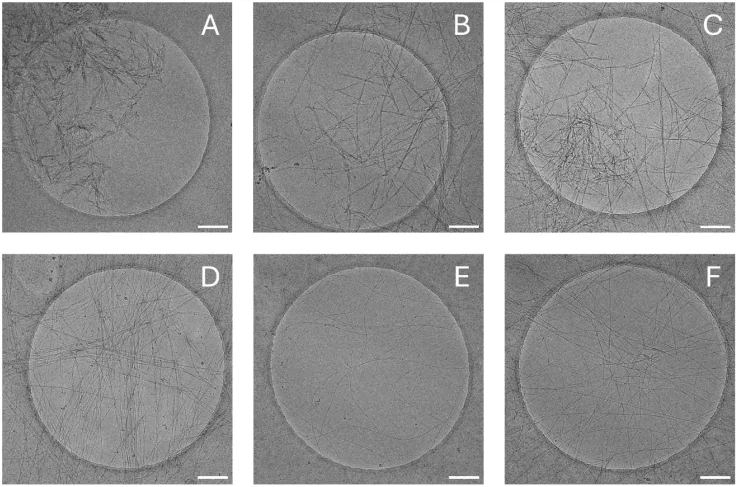
Cryo-EM images of hIAPP variant fibrils matured at pH 7.4, phosphate buffer 10 mM (A,B,C) and at pH 5.3, acetate buffer 30 mM (D,E,F), 37 °C, 30 μM concentration: wild type (A,D), tetra (B,E) and penta (C,F). The scale bar is 200 nm in size.

We also evaluated images of amyloid fibrils matured at acidic pH values. So far, there have been several reports of a wt hIAPP’s inability to form proper fibrils under acidic conditions.^23,25^ In our investigations, we readily found long fibrils that were twisted into various higher-order structures (Figure 7D-F). In the tetra hIAPP variant, which displayed the fastest aggregation rate, it was more challenging to find fibrils under the selected preparation conditions (Figure 7E) but the few fibrils that were found displayed the greatest homogeneity among all variants. The penta variant, having an aggregation rate similar to that of wt hIAPP, also showed a comparable fibrilization pattern (Figure 7F).

## Discussion

Thioflavin T fluorescence measurements provide a macroscopic kinetic readout of amyloid formation and, in this study, serve as a guide for identifying conditions that favor or disfavor productive fibril growth, which is then examined in mechanistic detail using complementary techniques. While kinetic analyses provide insight into the dominant aggregation regimes, the CD spectroscopic and mass-spectrometric data indicate that hIAPP aggregation proceeds through a conformationally heterogeneous landscape that is strongly modulated by both the pH and peptide concentration. In particular, the CD spectra often deviate from the canonical antiparallel β-sheet signature of mature hIAPP fibrils, instead reflecting a complex superposition of β-sheet, random-coil, and α-helical contributions, which could serve as intermediate states on the fibrilization pathway (Figure 4D).^13,41–43^ This observation suggests that the detected β-sheet signal does not solely report on the accumulation of mature fibrils but may partially originate from transient and kinetically stabilized conformational states populated under conditions of enhanced intermolecular interaction (Figure 6).

Previous structural studies have shown that Phe23 transiently engages in β-sheet interactions during the early stages of assembly before undergoing conformational rearrangement into a more disordered loop region in the mature fibril architecture.^44–46^ In this context, stabilization of the β-sheet structure at early stages—whether through extensive fluorination, elevated peptide concentration, or both—may reduce the conformational flexibility required for productive fibril growth. Thus, the higher fraction of β-sheet formation in our molecular dynamics simulations negatively correlated with the aggregation rate of a specific variant (Figure 2A,B and Figure 6). Conversely, conditions that preserve access to conformationally labile intermediates appear to promote efficient aggregation. Consistent with this view, correlation of secondary-structure content with aggregation kinetics reveals that increased accessibility of α-helical conformations at the early aggregation stages is associated with faster fibrilization at acidic and neutral pH (Figure 2A,B and Figure 6), in line with the established role of α-helical states as productive intermediates in hIAPP amyloidogenesis.^13,41–43,47^

The oligomeric distributions detected by ion mobility–mass spectrometry further support a conformational-preference model. Previous work has proposed that early hIAPP oligomers are stabilized primarily through β-strand-mediated interfaces and that subtle changes in the balance between ordered and disordered interactions can strongly influence aggregation outcomes.^45^ The enhanced β-sheet propensity determined for tetra- and penta-fluorinated variants at neutral pH correlates with the increased population of higher-order oligomers observed for these peptides (Figures 6, 3), suggesting that early stabilization of β-structured assemblies may bias the system toward aggregation regimes with reduced monomer dependence. Importantly, such assemblies need not be irreversibly off-pathway; rather, their accumulation may kinetically limit access to conformations that efficiently engage in elongation or secondary nucleation.

A similar principle has been observed in studies of macromolecular crowding, where the presence of inert crowders, such as bovine serum albumin (BSA) has been shown to slow hIAPP aggregation despite increased effective concentrations.^48^ In these systems, crowding stabilizes nonproductive intermolecular contacts, leading to delayed fibril formation. The high-concentration effects observed here may reflect an analogous mechanism in which enhanced intermolecular interactions favor conformational states that are structurally ordered yet poorly suited for productive fibril growth.

Taken together, these findings support a model in which productive hIAPP fibril formation is governed by pH- and concentration-dependent conformational preferences rather than by a single invariant aggregation pathway. Chemical perturbation by fluorination of Phe23 and environmental modulation through pH and concentration cooperatively reshape the accessible conformational landscape, determining whether aggregation proceeds efficiently or becomes self-limited by the stabilization of alternative assemblies. This framework reconciles the coexistence of β-sheet signatures with slowed aggregation kinetics and underscores the importance of probing amyloid formation across extended concentration regimes to fully capture the mechanisms governing hIAPP self-assembly.

## Conclusions

This study refines the understanding of the aggregation mechanism of the human islet amyloid polypeptide by revealing how productive fibril formation emerges from pH- and concentration-dependent conformational preferences rather than from a single invariant aggregation pathway. Phe23 emerges as a dynamic molecular switch that reports on the interplay between the intrachain conformational flexibility and interchain association required for efficient fibril growth. Modulation of His18 protonation reshapes these requirements by altering both intrachain conformational flexibility and interchain electrostatic interactions, leading to a distinct sequence-dependent aggregation response. In the wild-type peptide and minimally fluorinated variants, this balance can be achieved under both pH conditions, enabling Phe23 to be accommodated within β-sheet interfaces and allowing aggregation to proceed along the canonical nucleation–elongation mechanism, although enhanced intermolecular interactions at neutral pH can render fibrilization self-limited. Increased fluorination likely enhances intermolecular interactions, amplifying sensitivity to pH- and concentration-dependent conformational constraints and leading to divergent aggregation behavior of the tetra- and penta-fluorinated variants. These results highlight the potential of subtle stereoelectronic modifications to modulate amyloid aggregation pathways, providing a basis for the rational design of sequence-specific aggregation modulators.

## Materials and Methods

### General Methods

HRMS measurements were conducted on an Agilent 6220 ESI-TOF MS instrument (Agilent Technologies). For analysis, MassHunter Workstation Software Version B.02.00 (Agilent Technologies) was used. MS and NMR spectra were analyzed by using MestReNova 14.2. Fmoc-L-amino acids were purchased from Carbolution (St. Ingbert, Germany). Pseudoproline building blocks were purchased from IRIS Biotech (Marktredwitz, Germany). Fluorinated Fmoc-L-amino acids were purchased from commercial sources as follows: Fmoc-Phe­(4-F)–OH, Fmoc-Phe­(3,5-diF)–OH (ABCR, Karlsruhe, Germany), and FmocPhe-(2,3,4,5,6-pentaF)–OH (Fluorochem Limited, Derbyshire, England. Fmoc-Phe­(2,3,5,6-tetraF)–OH was synthesized according to literature.^49^

**Peptide synthesis** was conducted on a microwave-assisted Liberty Blue (CEM, Matthews, NC, USA) peptide synthesizer using standard fluorenylmethoxycarbonyl (Fmoc) chemistry with diisopropylcarbodiimide (DIC) as the activating agent and ethyl 2-cyano-2-(hydroxyamino)­acetate (Oxyma Pure) as additive. The approach decreasing aggregation through the synthesis with the pseudoproline building blocks integration for the 27–28 LS and 8–9 AT residues was used.^50,51^ Peptides were obtained in 0.05 and 0.1 mmol scales with Rink Amide ProTide Resin (LL) resin (0.19 mmol/g loading) from CEM. A 5-fold excess of amino acid (carbolution) was used in each coupling reaction. Details of specific cycle choice throughout the sequence and the corresponding parameters can be found in the Supporting Information (Figure S12-S15). All peptides were cleaved from the resin using a mixture of TFA/TIPS/H_2_O/EDT (94:1:2.5:2.5) for 2 h at room temperature. Crude peptides were precipitated in cold diethyl ether and centrifuged. The supernatant was discarded, and the pellet was washed twice by repeating this procedure. The final precipitate was lyophilized.

The workup was based on the previously established protocols.^50–52^ To obtain the physiologically relevant form of the hIAPP variants, the crude peptides were dissolved in 1,1,1,3,3,3-hexafluoro-2-propanol (HFIP) at a concentration of 10 mg/mL and oxidized to form the disulfide bridge Cys_2_-Cys_7_ by the addition of 5 mol equiv of iodine (I_2_) to 1 equiv of peptide, calculated based on the crude peptide mass assumed pure. Oxidation was completed within 20–30 min, as confirmed by analytical HPLC (gradient of eluent B from 20% to 60% over 18 min) and later was confirmed by high-resolution mass spectrometry. The HFIP solution was filtered through a 0.2 μm PTFE syringe filter and concentrated 5-fold by solvent evaporation under a stream of nitrogen. The resulting solution was diluted 20-fold in 30% acetic acid to reach an approximate concentration of 2 mg/mL. The samples were then filtered through a 0.45 μm PES syringe filter and injected into the preparative HPLC system for purification.

### HPLC Analysis and Purification

Analysis of peptides was performed on the Primaide DAD system (VWR/Hitachi, Germany) operating on a low-pressure gradient with a Kinetex C18 column (5 μm, 100 Å, 250 × 4.6 mm, Phenomenex, Torrance, CA, USA) and a SecurityGuard cartridge kit containing a C18 cartridge (4 × 3.0 mm, Phenomenex, Torrance, CA, USA) as precolumn. As eluents, water (eluent A) and acetonitrile (eluent B), both containing 0.1% (v/v) TFA, were used at a flow rate of 1 mL/min. The preliminary analysis of crude peptides was carried out with a linear gradient of 10–80% eluent B over 18 min with detection at a wavelength of 220 nm. The gradient 20–60% eluent B over 18 min was used for the analysis of the oxidation reaction and further fraction control of purified peptides. The data were analyzed using an OpenLab EZChrom Elite (VA.04.10, Agilent).

Purification was carried out on the system of Nexera Prep from Shimadzu (Duisburg, Germany) comprising a system controller SCL-40, UV–vis detector SPD-40 with a flow cell 40UV of 10 mm path length, a preparative liquid chromatograph LC-20AP including a mixing chamber, a prep quaternary valve FCV-200AL, and a liquid handler LH-40. A Kinetex C18 column (5 μm, 100 Å, 250 × 21.2 mm; Phenomenex, Torrance, CA, USA). The same eluents as those used for the analytical HPLC were used at a flow rate of 15 mL/min. Peptides were purified using a linear gradient of 20–60% eluent B over 35 min, with the first 2.5 min of injection with detection at a wavelength of 220 nm. The data were analyzed by using LabSolutions (V5.117) software. The product peaks were fractionated and analyzed via analytical HPLC with the gradient 20–60%, 18 min and characterized by mass spectrometry (Figures S16-S20). The pure fractions (>95%) were combined and lyophilized.

### Thioflavin T Fluorescence Assay

As a general procedure, stock solutions of hIAPP variants were prepared by dissolving the purified peptides in HFIP to the approximate concentration of 2 mg/mL and further sonicated for 5 min to dissolve all preformed aggregates and filtered through a 0.2 μm PTFE syringe filter after that. The concentration was determined by measurements of absorbance at 280 nm in 6 M Gdn*HCl solution and calculations based on the literature extinction coefficient. Aliquots of these stock solutions were transferred directly into the BRAND microplate (size: 96 wells, color: black; Sigma-Aldrich) dried and then redissolved in the corresponding buffer—sodium acetate 30 mM, pH 5.3 or simple phosphate buffer solution 10 mM, pH 7.4, containing 20 μM ThT. The buffer containing ThT was filtered through a PES syringe filter with 0.2 μm pore size. After dissolution, the samples in the well-plate were sonicated for 15 s and sealed to prevent evaporation. The plate was placed in an InfiniteM200 plate reader with a constant incubation temperature of 37 °C (Tecan Nordic AB, Molndal, Sweden). ThT fluorescence (λex = 420 nm, λem = 485 nm, Z-position: 15173 nm [manual], gain: 80 [manual], lag time: 0 μs, integration time: 20 μs) was measured every 10 min.

### CD Spectroscopy for Secondary-Structure Analysis

CD spectra were taken on a JASCO-810 spectropolarimeter (JASCO Deutschland GmbH, Pfungstadt, Germany), connected to the JASCO PTC-423S Peltier element and a HAAKE WKL water recirculator (Thermo Electron GmbH, Karlsruhe, Germany) for temperature control. Quartz Suprasil cuvettes (Hellma Analytics, Mühlheim, Germany) with path lengths of 1, 2, and 10 mm were used. Measurements were performed in the UV range of 190–250 nm with the accumulation of 3 scans and baseline correction utilizing corresponding blank buffers. Data were collected using the software JWS-510-J-800 Spectra Manager version 2 (JASCO Deutschland GmbH, Pfungstadt, Germany).

### Cryo-Transmission Electron Microscopy

Cryo-EM was used to confirm the presence of amyloid fibrils. The materials were collected after the time corresponding to the plateau phase in the aggregation process (2 days for pH 7.4; 7 days for pH 5.3). 3.8 μL were applied to perforated carbon film-covered microscopical grids (200 mesh, R1/4 batch of Quantifoil, MicroTools GmbH, Jena, Germany), which were cleaned with chloroform and hydrophilized by 60 s glow discharging at 8 W in BALTEC MED 020 device (Leica Microsystems, Wetzlar, Germany) before use. The grids were then plunge-frozen in a Vitrobot Mark IV (Thermo Fisher Scientific Inc., Waltham, Massachusetts, USA) using liquid ethane as a cryogen. The samples were transferred to a Talos Arctica electron microscope equipped with a high-brightness field-emission gun (XFEG) operated at 200 kV acceleration voltage. Micrographs were acquired on a Falcon 3 direct electron detector (Thermo Fisher Scientific Inc., Waltham, Massachusetts, USA) at nominal magnifications of 28,000×.

### ^19^F Solution NMR

All ^19^F NMR data were recorded on a 400 MHz JEOL ECS spectrometer equipped with a 40TH5/AT/FG2D probehead. The spectra were acquired by operating at a ^19^F resonance frequency of 376.13 MHz using a single-pulse experiment. Measurements were performed at 19.5 °C. Spectra were collected with a spectral width of 94.3 kHz and an acquisition time of 2.22 s, using 262144 data points. A total of 2048 or 8192 (for the mono variant) scans were accumulated with a relaxation delay of 0.10 s and a pulse width of 10.9 μs. The receiver gain was set automatically in a range of 18.0 to 24.0. Data were processed using zero filling to a final spectral size of 209716 points prior to Fourier transformation. The spectra for each variant were recorded at least twice, and the reproducibility of chemical shifts was verified. The signal of the TFA counterion was used as an internal chemical shift reference, and all of the ^19^F NMR spectra were referenced accordingly.

### MD Simulations

As a starting point for the simulations, we used the solution NMR structure of oxidized and amidated human IAPP (residues 1–37; PDB ID: 5MGQ).^40^ To probe the role of aromatic modifications, variants with tetra- and pentafluorinated phenylalanines at position 23 were generated. Parameterization of the fluorinated side chains was performed with the CHARMM General Force Field (CGenFF)^53^ using the Force Field Toolkit (ffTK) implemented in VMD.^54^ Partial atomic charges were optimized based on ab initio calculations at the BLYP/SVP level using ORCA 6.1.^55^ A single peptide was placed in a cubic box of explicit TIP3P water molecules and neutralized with counterions, and NaCl was added to reach a final ionic strength of 0.15 M. For the single-peptide simulations, three systems were considered: wild-type hIAPP, tetrafluorinated Phe23, and pentafluorinated Phe23. Each system was simulated for two different protonation conditions of histidine residues: fully protonated (to mimic acidic pH) and neutral (to mimic physiological pH). Energy minimization was followed by equilibration runs under NVT and NPT ensembles. All simulations were carried out using GROMACS 2024.2.^56^ For the single-peptide systems, production runs of 1 μs were performed for each peptide variant under both histidine protonation states. To investigate aggregation, systems of 10 peptides were constructed using PACKMOL^57^ in cubic boxes of 120 × 120 × 120 Å^3^, with random initial orientations and positions. These multimeric systems (wild-type, tetrafluorinated, and pentafluorinated hIAPP) were simulated again for two different histidine protonation states, as described above. Peptide interactions were described using the CHARMM36m force field, while water molecules were modeled with TIP3P. Long-range electrostatics were treated with the particle mesh Ewald method (cutoff 12 Å). van der Waals interactions were smoothly shifted to zero between 10 and 12 Å. All covalent bonds involving hydrogen atoms were constrained using the LINCS algorithm, allowing a 2 fs time step. Temperature was maintained at 310 K using the velocity-rescaling thermostat,^58^ and pressure was maintained at 1 bar using the Parrinello–Rahman barostat.^59^ Trajectory analyses were performed using MDAnalysis^60^ and in-house Python scripts. We monitored the secondary structure propensity, solvent-accessible surface area (SASA), radius of gyration, and intermolecular contact maps. Per-residue SASA was computed with the Shrake–Rupley algorithm (probe = 1.4 Å),^61^ per frame and chain, and then time-averaged; distributions were also recorded. Phe23 refers to the aromatic heavy atom in residue 23.

## Supplementary Material



## Notes

ThT fluorescence kinetics and scaling analysis, pyrene fluorescence assay, native mass spectrometry, circular dichroism spectra, molecular dynamics simulations, synthesis protocols, analytical HPLC, and mass spectrometry characterization of hIAPP variants (PDF)
