# Supplementary material for: Fine-Tuning hIAPP Amyloidogenesis: Probing Molecular Mechanisms via Fluorinated Core Substitutions
Source: ACS Bio Med Chem Au. 2026 May 5;6(4):381–92. doi: 10.1021/acsbiomedchemau.6c00061 (PMC13495020; doi:10.1021/acsbiomedchemau.6c00061)
Supplement: Supplementary file 1 [file bg6c00061_si_001.pdf]

Supporting Information

Fine-Tuning hIAPP Amyloidogenesis: Probing Molecular Mechanisms via Fluorinated Core Substitutions

Artem Pavlov†, Hossein Batebi‡, Nicklas Österlund†, Kevin Pagel†, Boris Schade⊥, Mario Schubert, Roland R. Netz‡, Beate Koksch *†

† Department of Chemistry and Biochemistry, Freie Universität Berlin, Arnimallee 20, 14195 Berlin, Germany
‡ Department of Physics, Freie Universität Berlin, Arnimallee 14, Berlin 14195, Germany
⊥Research Center of Electron Microscopy, Freie Universität Berlin, Berlin, Germany

Table of Contents

Figure S1. Normalized ThT fluorescence intensity versus time graphs with a fitting 2
Figure S2-3. Scaling exponent calculations for the ThT kinetic experiments... 4
Figure S4. Pyrene fluorescence experiments. 6
Figure S5. Native mass spectrometry analysis of the IAPP wild type and the penta variant............ 6
Figure S6-7. Circular dichroism spectra of hIAPP variants 7
Figure S8-11. Molecular-dynamics simulations data 8
Figure S12-15. Protocols used for the synthesis of hIAPP variants. 11
Figure S16-20. Analytical HPLC chromatogram and MS spectrum of hIAPP variants 15

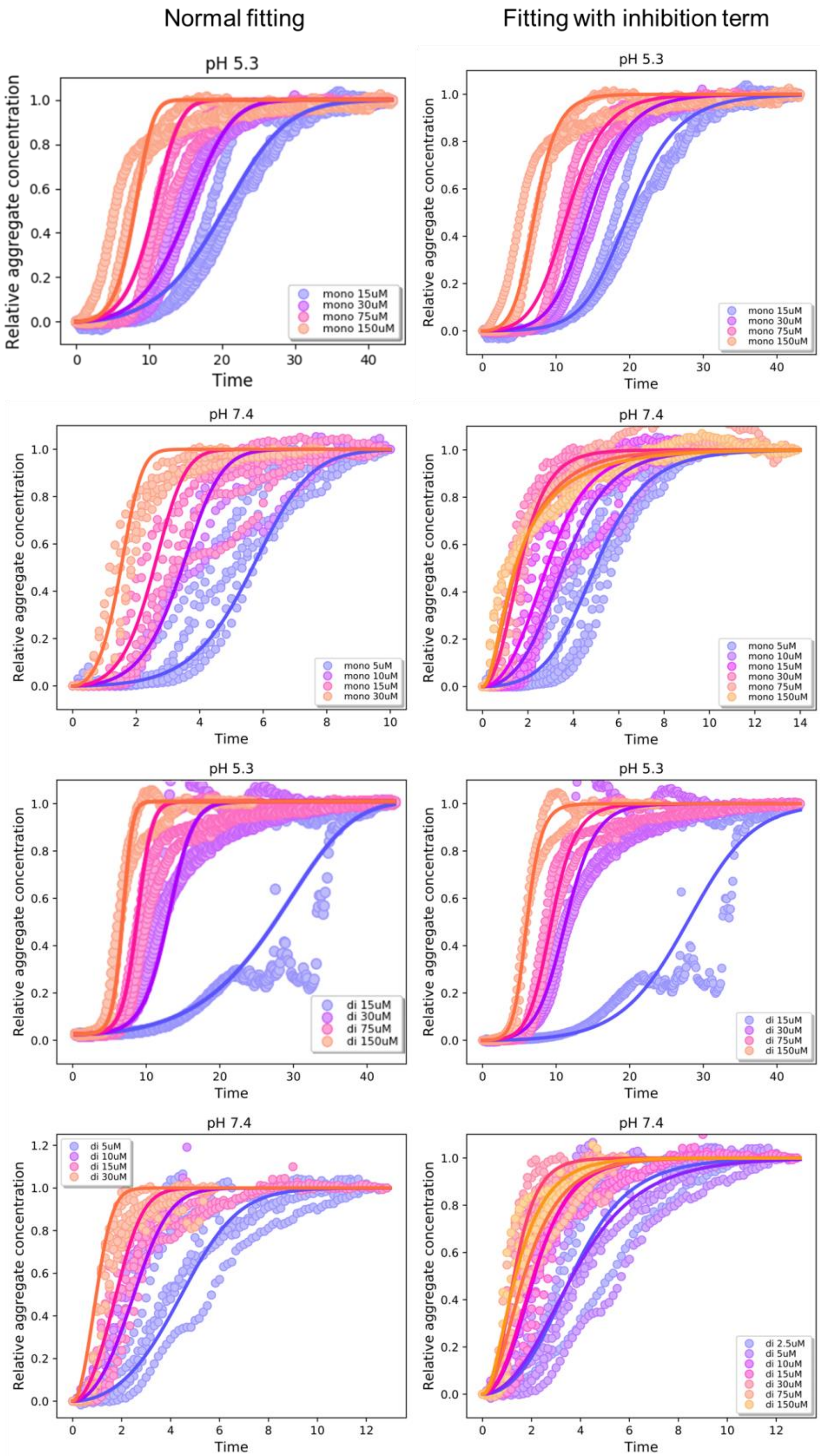

Figure S1. Normalized ThT fluorescence intensity versus time for 3-6 replicates at each concentration with a fitting used a model including primary nucleation, multi-step secondary nucleation and elongation (left) and with the additional inhibition term (right).

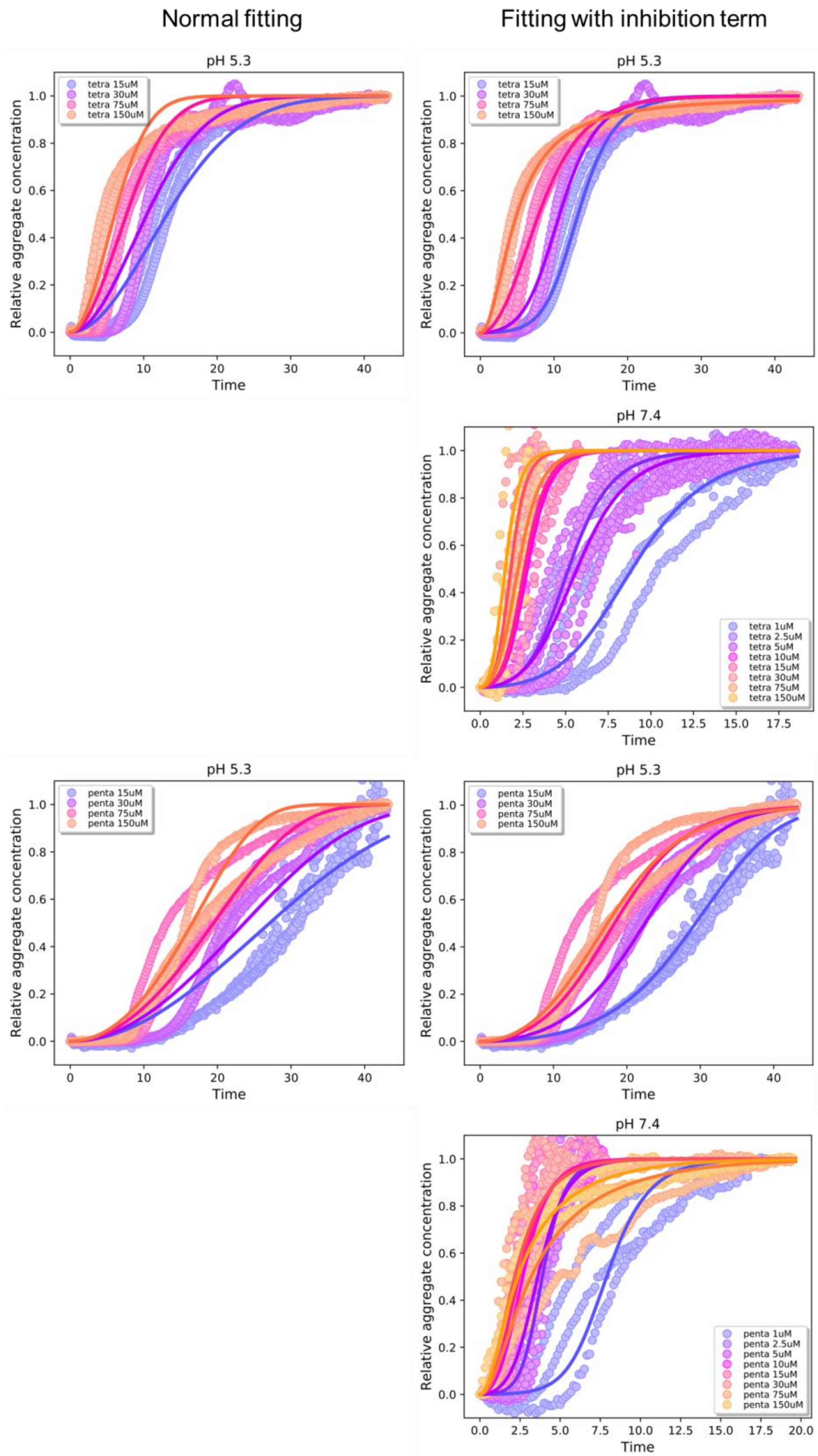

Figure S1 (continuation). Normalized ThT fluorescence intensity versus time for 3-6 replicates at each concentration with a fitting used a model including primary nucleation, multi-step secondary nucleation and elongation (left) and with the additional inhibition term (right).

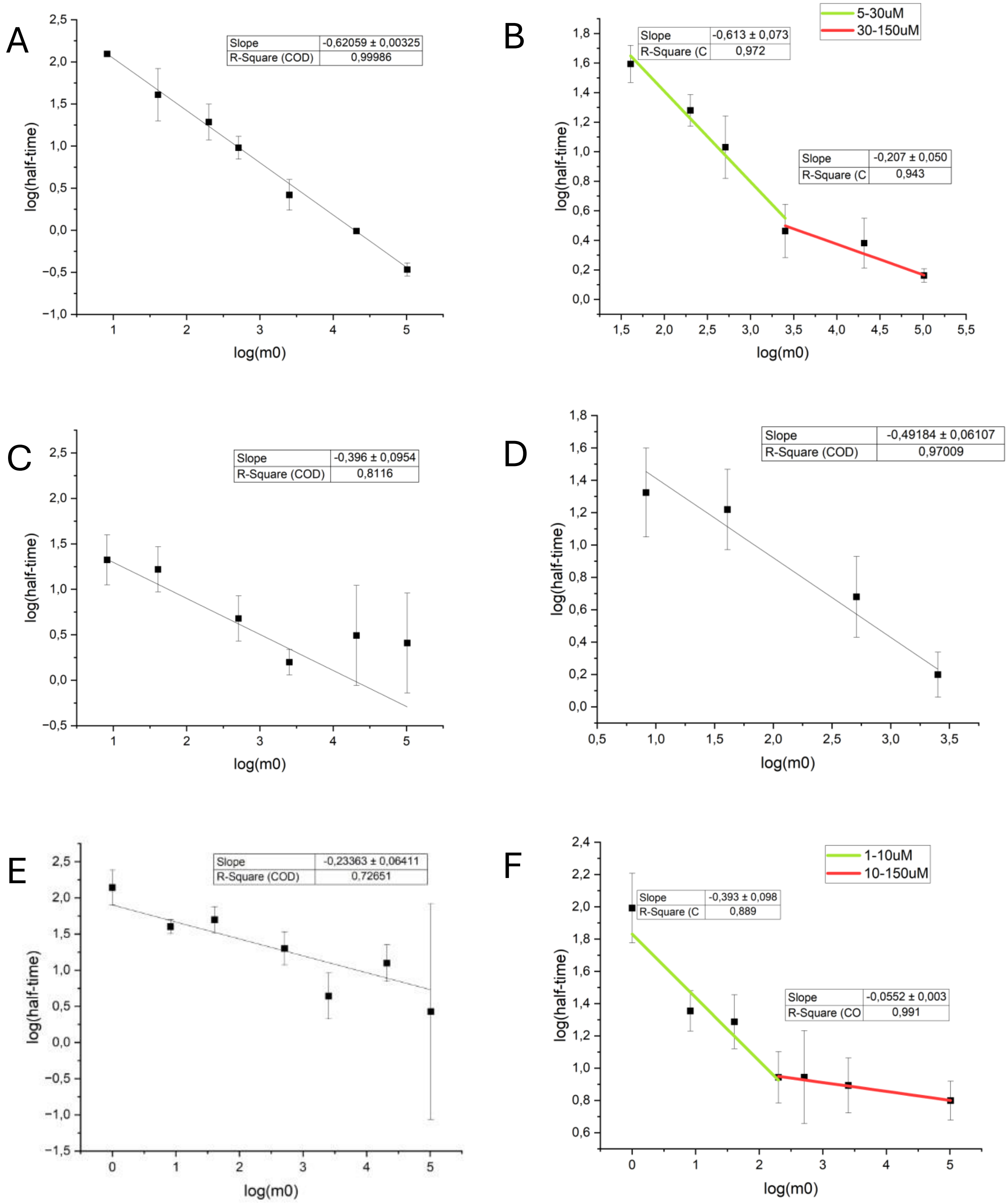

Figure S2. Scaling exponent calculations for the ThT kinetic experiments at pH 7.4: wt (A), mono (B), di with the whole set of experiments, 2.5-150 μM (C), di experiments within 2.5-30 μM concentration, tetra (E), penta (F).

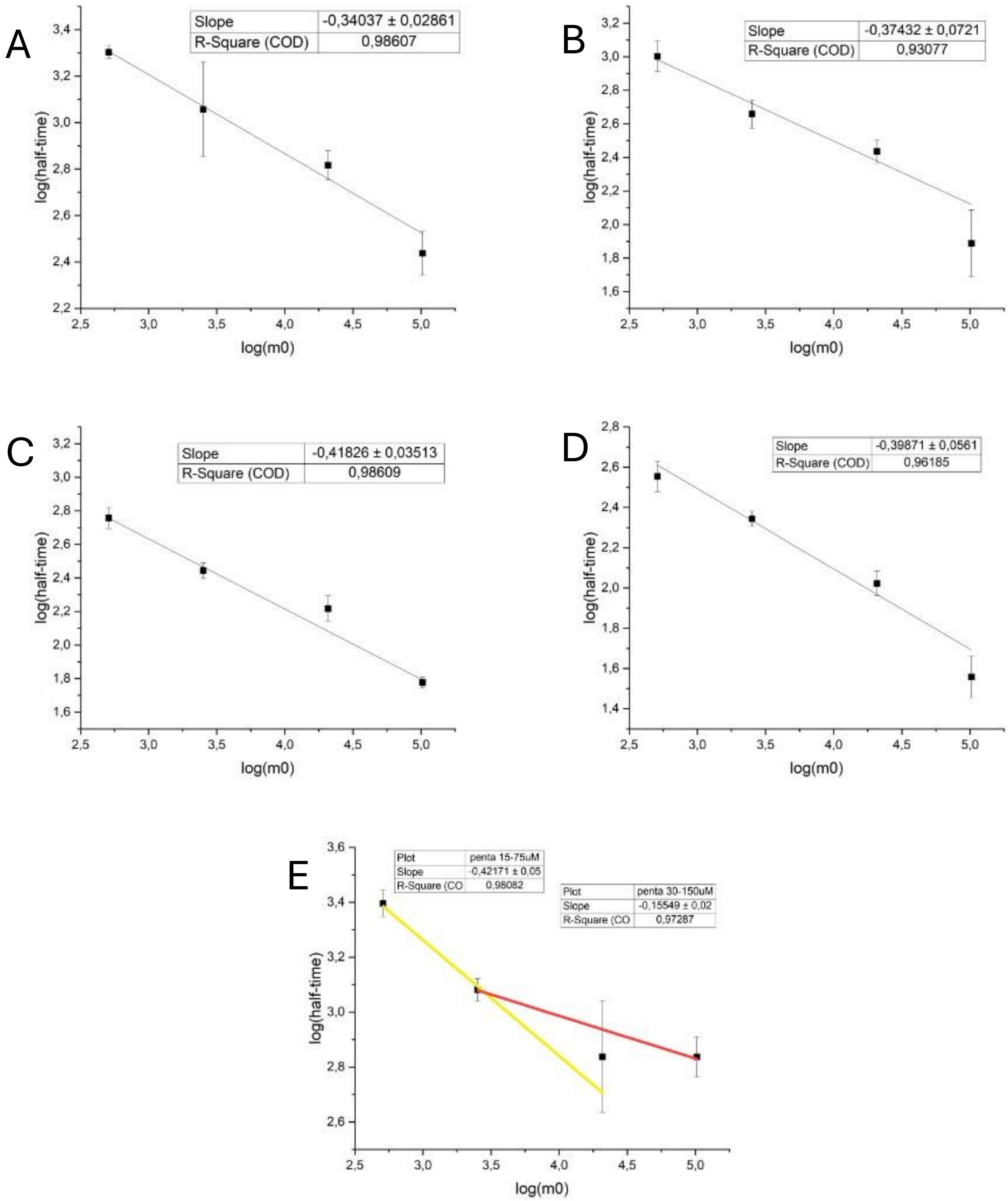

Figure S3. Scaling exponent calculations for the ThT kinetic experiments at pH 5.3: wt (A), mono (B), di (C), tetra (D), penta (E).

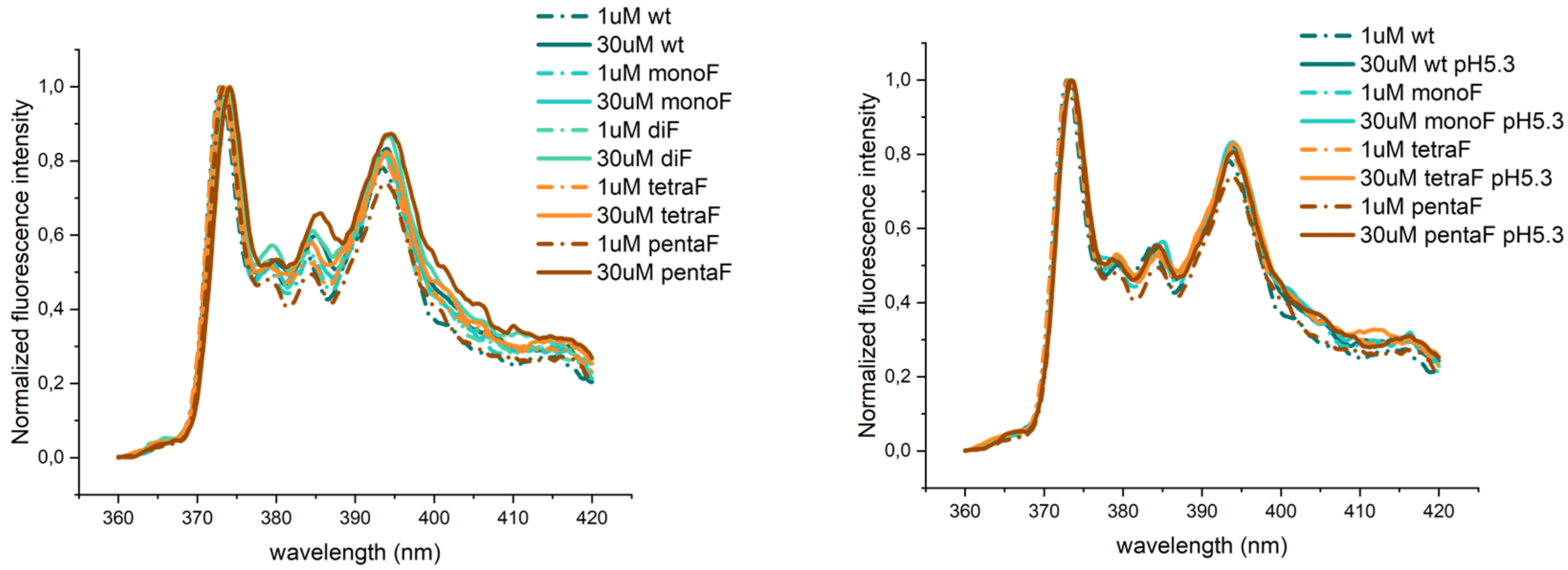

Figure S4. Pyrene fluorescence experiment. Extreme points of titration with the peptides (1 and 30 μM): left – for pH 7.4 (phosphate buffer), 1 and 30 μM, and right – pH 5.3 (acetate buffer), 30 μM, with the spectra of pH 7.4, 1 μM included.

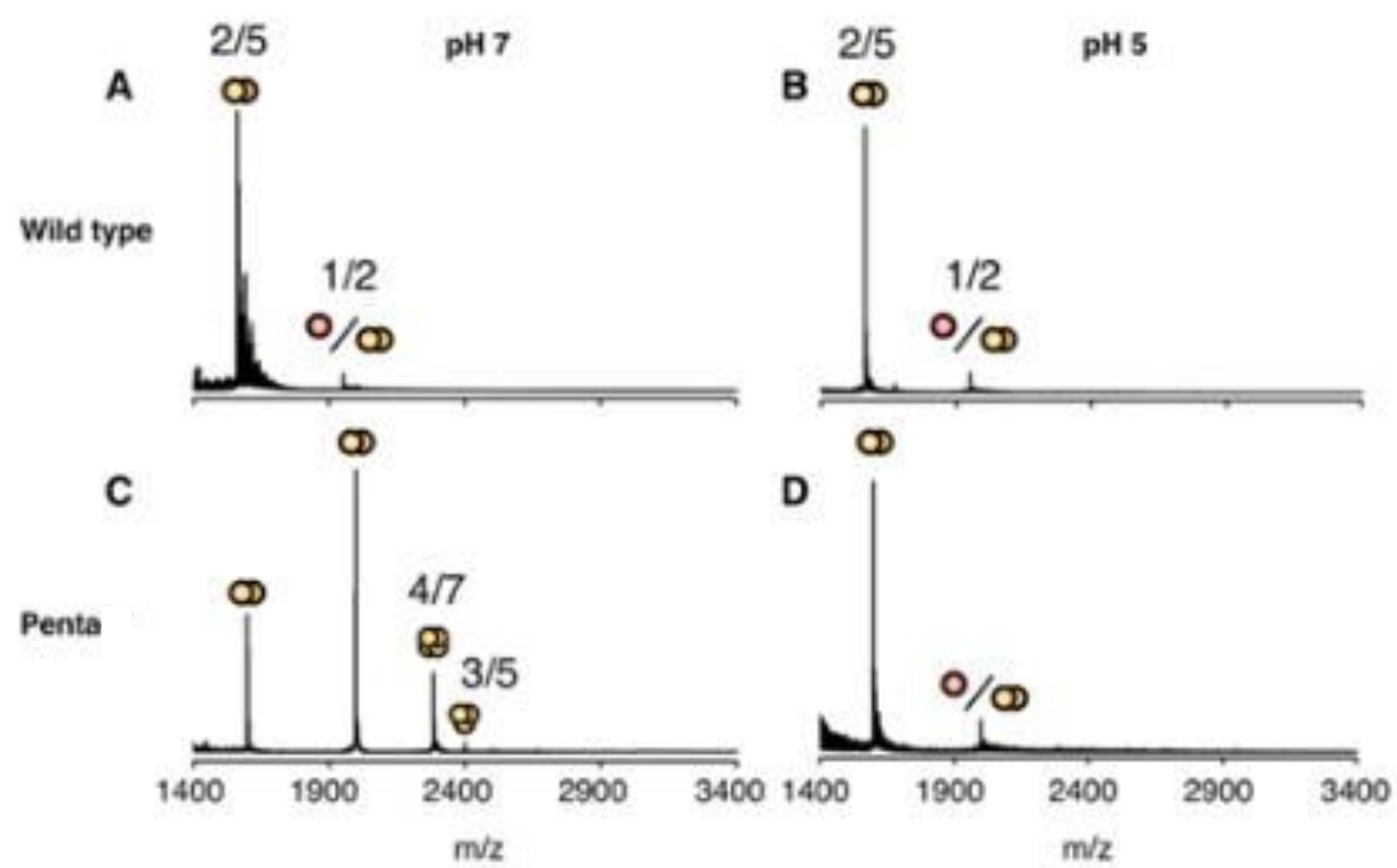

Figure S5. Native mass spectrometry analysis of the IAPP wild type (A,B) and the penta variant (C,D) in 200 mM ammonium acetate solution pH 7 (A,C) or acidified to pH 5 with acetic acid (B,D). Only the oligomeric region of the spectra are shown here.

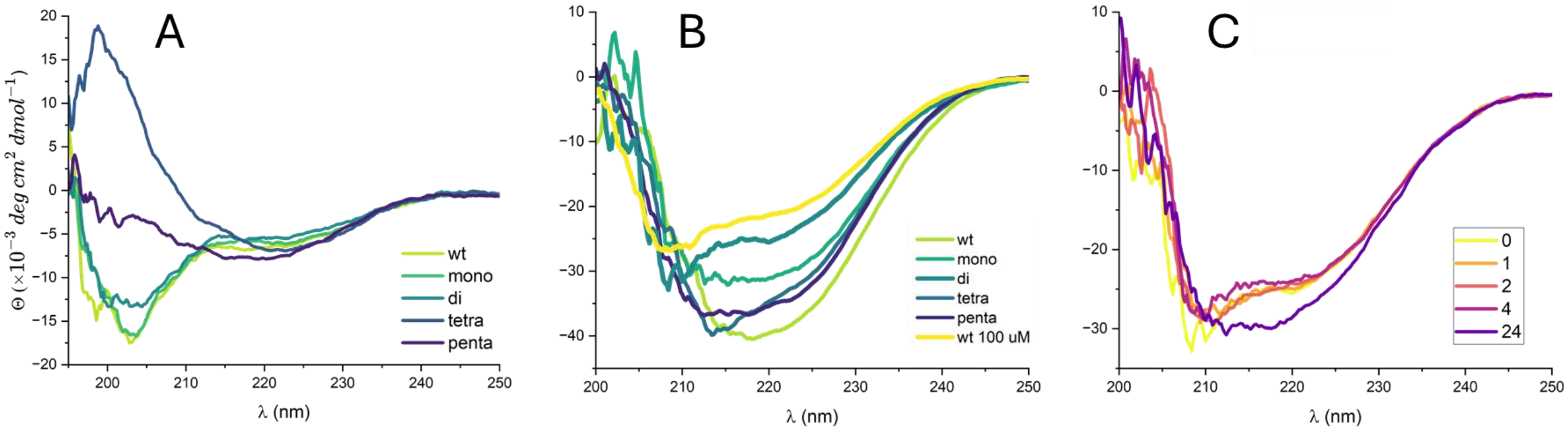

Figure S6. Circular dichroism spectra of hIAPP variants in acetate buffer (pH 5.3): (A) samples at a concentration of 30 μM incubated at room temperature for one week; (B) variants at 150 μM, along with an additional spectrum at 100 μM concentration, measured immediately after preparation. (C) Conformational transition monitoring of the di variant at 150 μM in acetate buffer (pH 5.3); the legend indicates the incubation time (h) for each spectrum.

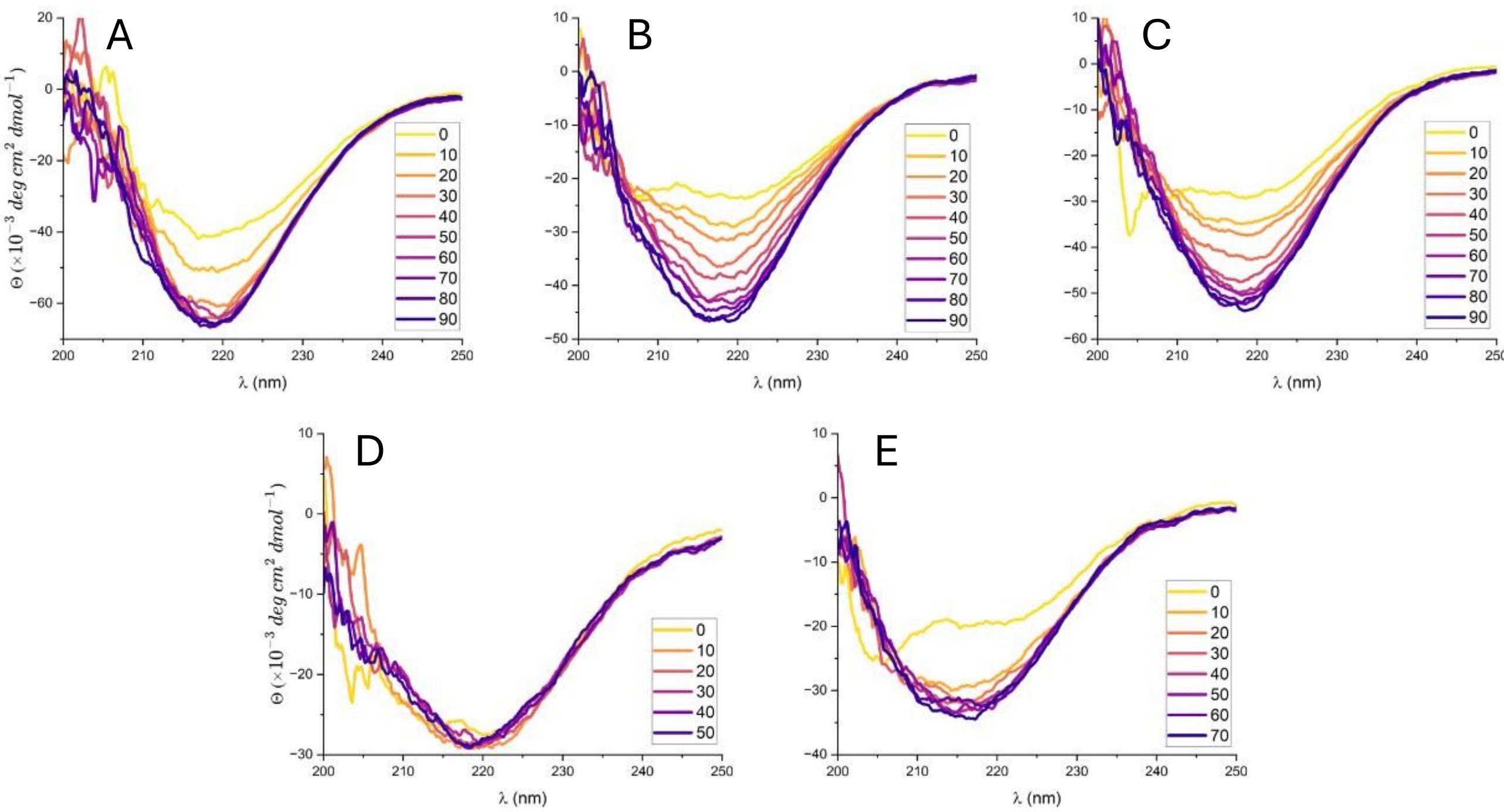

Figure S7. Circular dichroism spectra of hIAPP variants at a concentration of 150 μM in phosphate buffer (pH 7.4) during incubation at 37 °C: wt (A), mono (B), di (C), tetra (D), penta (E). The legend in each panel indicates the incubation time (min) for the corresponding spectrum.

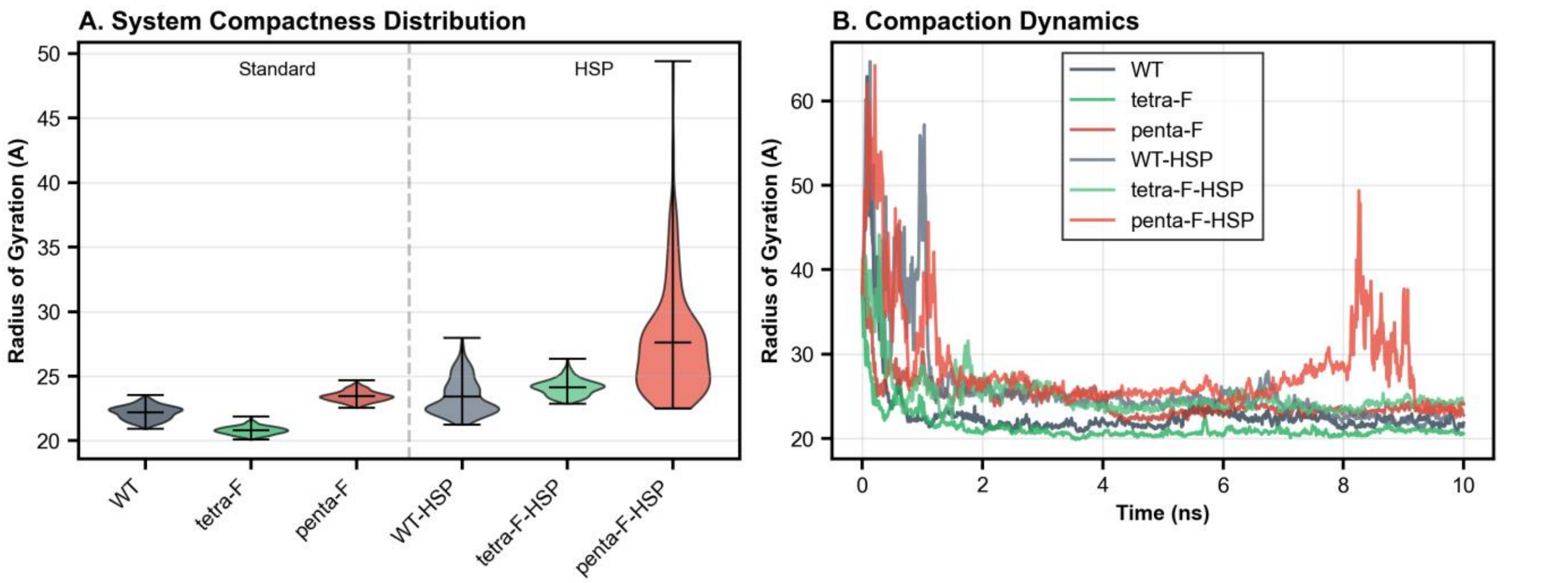

Figure S8. System compactness of multi-peptide assemblies from molecular-dynamics simulations.
(A) Violin plots of the radius of gyration (Rg) distributions for wild-type WT, tetrafluorinated (PTF), and pentafluorinated (PPF) assemblies under both neutral (Standard) and protonated histidine states (HSP). (B) Time evolution of Rg over the first 10 ns highlights differences in the rate and stability of chain compaction.

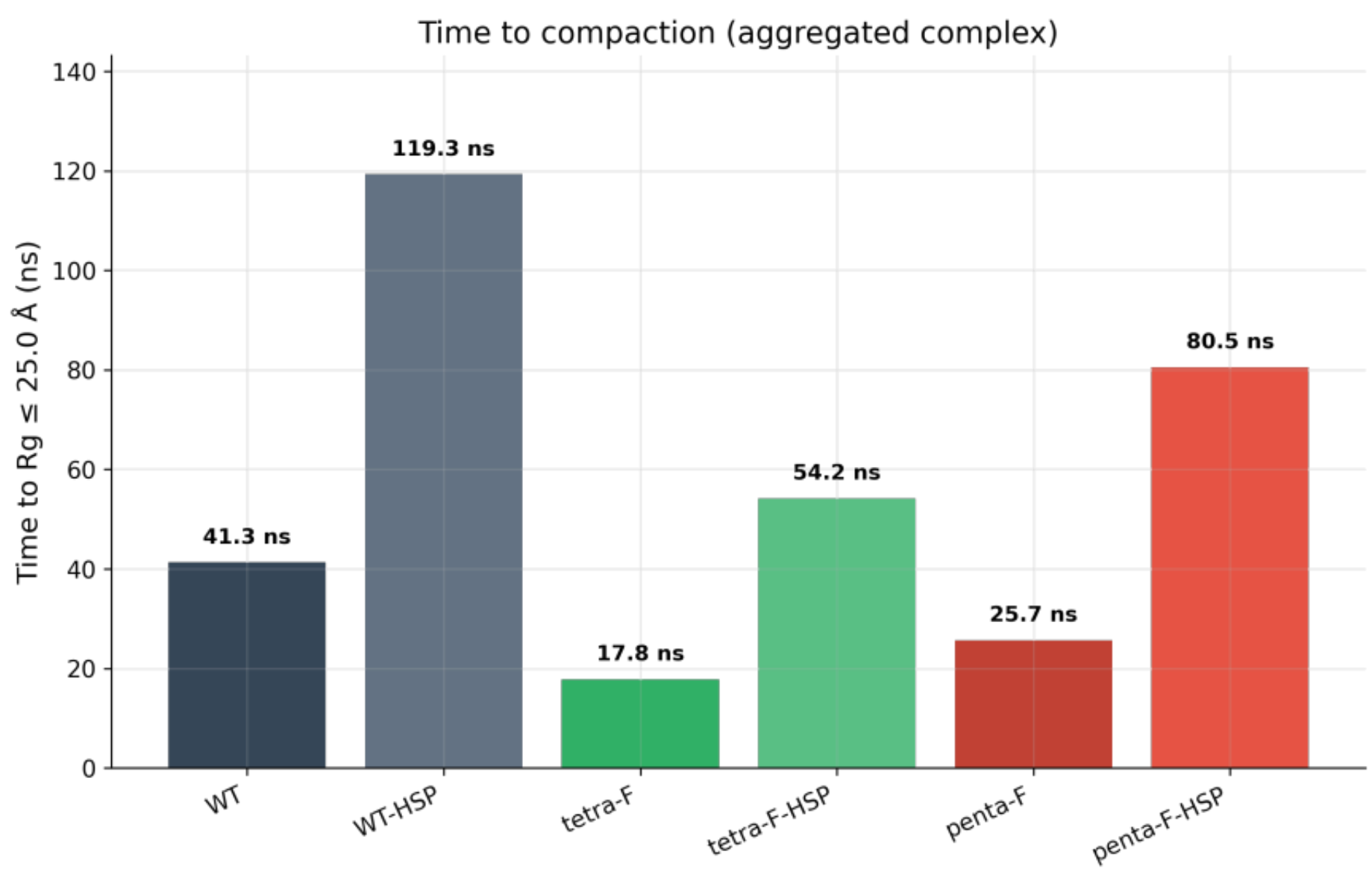

Figure S9. Timescale of oligomer compaction from molecular-dynamics simulations.
Time required for the 10-peptide systems to first reach a compact state, defined as Rg ≤ 25 Å. Under neutral conditions, tetra-fluorinated Phe23 compacts the fastest (17.8 ns), followed by penta-fluorinated Phe23 (25.7 ns), while wild type compacts more slowly (41.3 ns). Protonation of histidines (HSP) delays compaction across all systems, most dramatically in wild type (119.3 ns). Both fluorinated variants still compact faster than WT under acidic conditions, though the delay is significant (54.2 ns for tetra-fluorinated and 80.5 ns for penta-fluorinated Phe23).

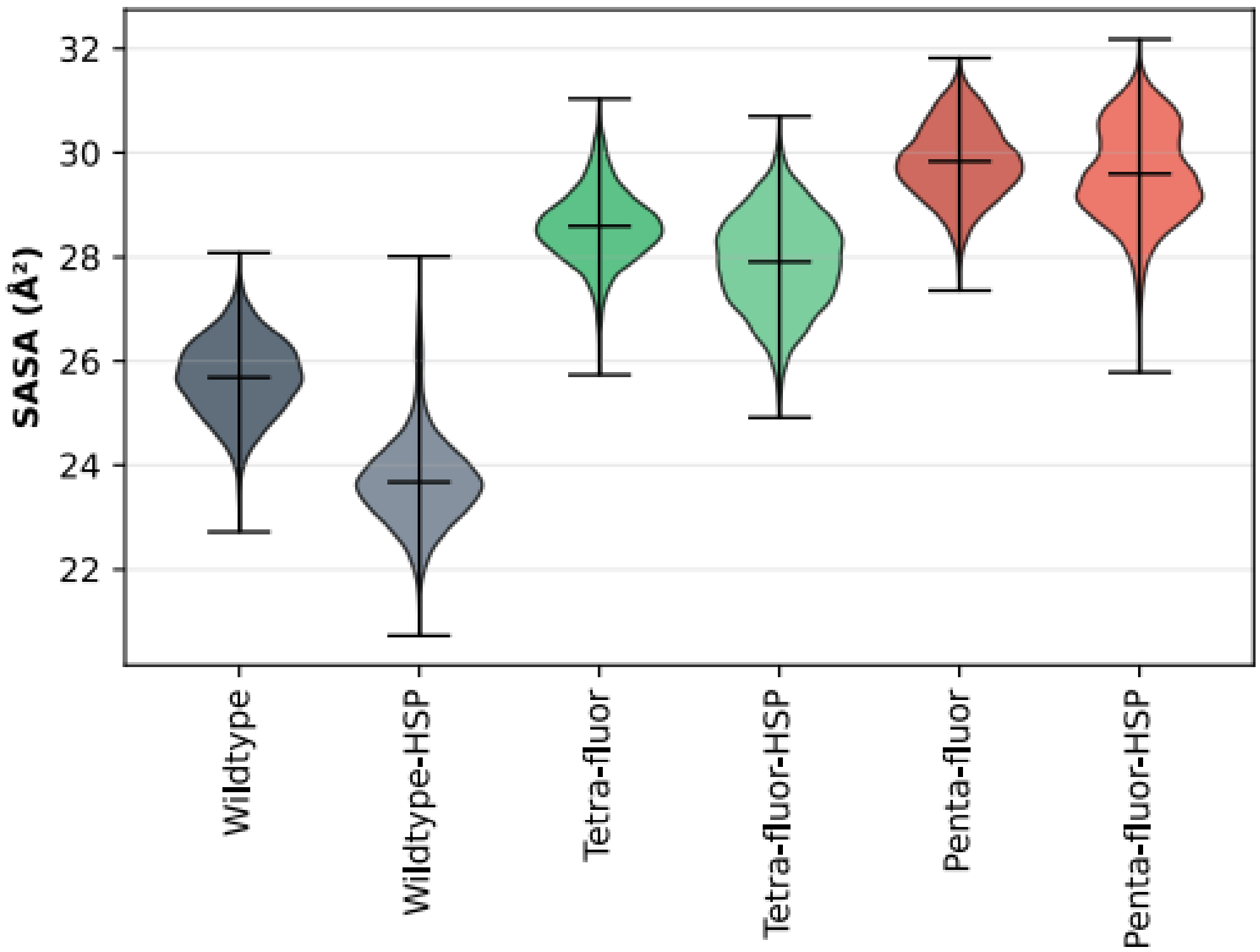

Figure S10. Solvent accessibility of Phe23 side chains from molecular-dynamics simulations.
Distribution of solvent-accessible surface area (SASA) of residue 23 across WT, tetra-fluorinated, and penta-fluorinated assemblies under neutral and protonated (HSP) conditions. Fluorination increases solvent exposure of Phe23, particularly for the penta-fluorinated variant, while protonation increases the variance of the distributions.

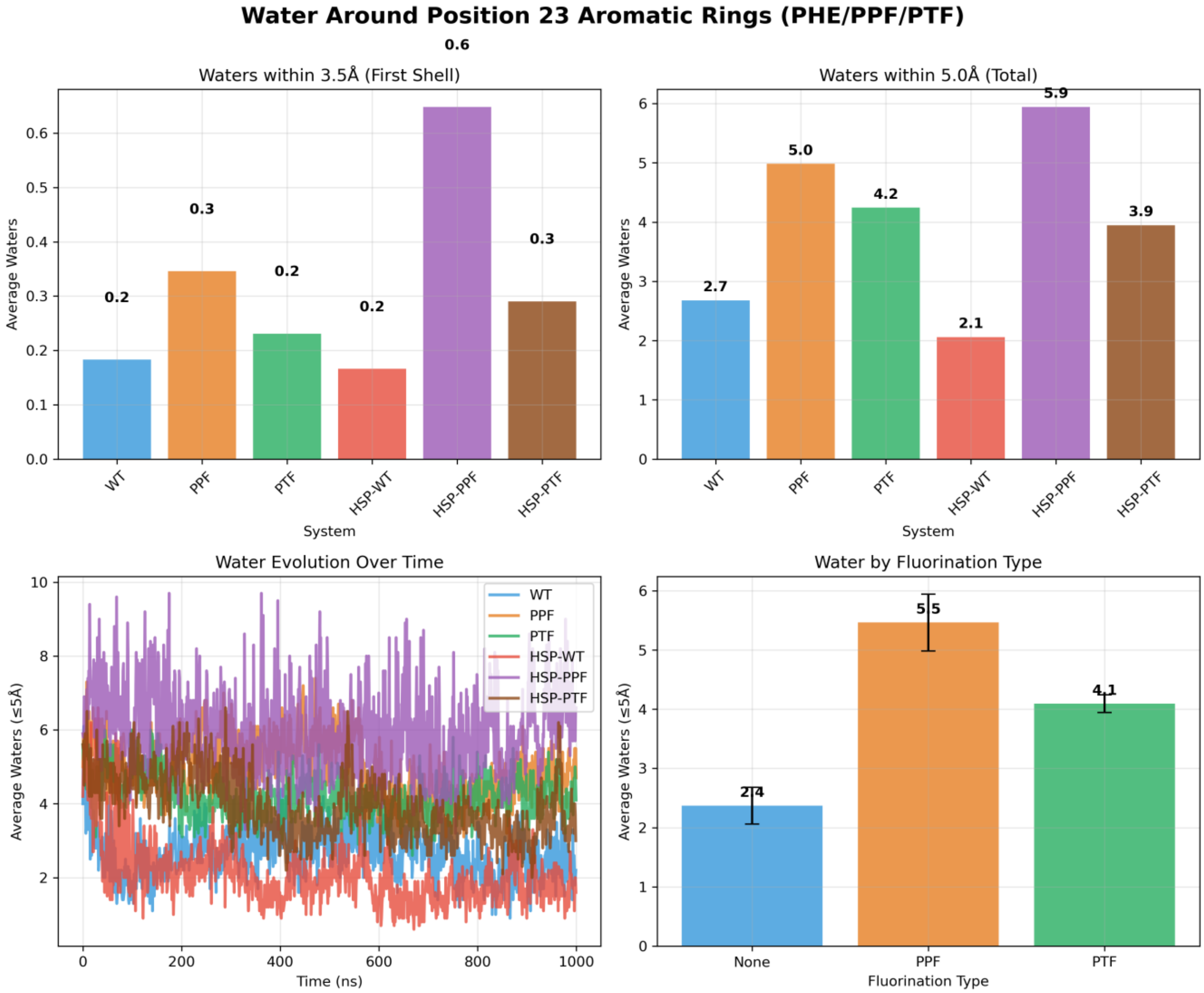

Figure S11. Hydration of Phe23 side chains from molecular-dynamics simulations.
(A) First shell (≤3.5 Å): mean number of waters within 3.5 Å of the Phe23 ring centroid.
(B) Total shell (≤5.0 Å): mean number of waters within 5.0 Å.
(C) Time evolution (≤5.0 Å): per-frame average number of waters over the first 1000 frames (≈ 1 µs) at stride = 1.
(D) By fluorination: comparison of hydration for no fluorination (WT, Phe23), tetra-fluorination (PTF), and penta-fluorination (PPF) at Phe23. Bars show the mean of the per-system trajectory means; error bars are ±1 SD across systems in the group.

Sequence: K C N T A T C 7 Q R L A N F L V H S S N N F G A I 5 S T N V G S N T Y - $CONH_2$

Resin	
**Name**	
**Scale**	0.05 mmol
**Loading**	0.19 mmol/g
**Type**	High-Swelling
**Preloaded**	No
**C-Terminus**	Amide
**Mass Required**	0.263 g

Cycles	
**Resin**	0.05-No Resin Swelling (HS)
**Final Deprotection**	0.05-Final Deprotection (HS)

Amino Acid	Specific Derivative	Cycle
Lys - K	Fmoc-Lys(Boc)-OH	0.05-Double Coupling (HS)
Cys - C	Fmoc-Cys(Trt)-OH	0.05-Double Coupling (HS)
Asn - N	Fmoc-Asn(Trt)-OH	0.05-Double Coupling (HS)
Thr - T	Fmoc-Thr(tBu)-OH	0.05-Double Coupling (HS)
Ala - A	Fmoc-Ala-OH	0.05-Double Coupling (HS)
Thr - T	Fmoc-Thr(tBu)-OH	0.05-Double Coupling (HS)
Cys - C	Fmoc-Cys(Trt)-OH	0.05-Double Coupling (HS)
EX7 - 7	External Amino Acid 7	0.05-Double 4min 90C Coupling (HS)
Gln - Q	Fmoc-Gln(Trt)-OH	0.05-Single 4min 90C Coupling (HS)
Arg - R	Fmoc-Arg(Pbf)-OH	0.05-Double Coupling (HS)
Leu - L	Fmoc-Leu-OH	0.05-Single 4min 90C Coupling (HS)
Ala - A	Fmoc-Ala-OH	0.05-Single 4min 90C Coupling (HS)
Asn - N	Fmoc-Asn(Trt)-OH	0.05-Single 4min 90C Coupling (HS)
Phe - F	Fmoc-Phe-OH	0.05-Single 4min 90C Coupling (HS)
Leu - L	Fmoc-Leu-OH	0.05-Single 4min 90C Coupling (HS)
Val - V	Fmoc-Val-OH	0.05-Single 4min 90C Coupling (HS)
His - H	Fmoc-His(Trt)-OH	0.05-Single 50°C 10 min Coupling (HS)
Ser - S	Fmoc-Ser(tBu)-OH	0.05-Single Coupling (HS)
Ser - S	Fmoc-Ser(tBu)-OH	0.05-Single Coupling (HS)
Asn - N	Fmoc-Asn(Trt)-OH	0.05-Single Coupling (HS)
Asn - N	Fmoc-Asn(Trt)-OH	0.05-Single Coupling (HS)
Phe - F	Fmoc-Phe-OH	0.05-Single Coupling (HS)
Gly - G	Fmoc-Gly-OH	0.05-Single Coupling (HS)
Ala - A	Fmoc-Ala-OH	0.05-Single Coupling (HS)
Ile - I	Fmoc-Ile-OH	0.05-Single Coupling (HS)
EX5 - 5	External Amino Acid 5	0.05-Single Coupling (HS)
Ser - S	Fmoc-Ser(tBu)-OH	0.05-Single Coupling (HS)
Thr - T	Fmoc-Thr(tBu)-OH	0.05-Single Coupling (HS)
Asn - N	Fmoc-Asn(Trt)-OH	0.05-Single Coupling (HS)
Val - V	Fmoc-Val-OH	0.05-Single Coupling (HS)
Gly - G	Fmoc-Gly-OH	0.05-Single Coupling (HS)
Ser - S	Fmoc-Ser(tBu)-OH	0.05-Single Coupling (HS)
Asn - N	Fmoc-Asn(Trt)-OH	0.05-Single Coupling (HS)
Thr - T	Fmoc-Thr(tBu)-OH	0.05-Single Coupling (HS)
Tyr - Y	Fmoc-Tyr(tBu)-OH	0.05-double deprotection, single coupling 4min 90C Coupling (HS)

Figure S12. Protocol used for the synthesis of wt hIAPP.

0.05-No Resin Swelling (HS)

Step	Operation	Parameters	Pause
1	Swell Resin	Main solvent volume: 10<br>Time: 10	False

0.05-Final Deprotection (HS)

Step	Operation	Parameters	Pause
1	Deprotection	ID: Standard Deprotection<br>Deprotection volume: 3	False
2	Wash	Main solvent volume: 4<br>Time: 5.0<br>Drain type: Default	False
3	Wash	Main solvent volume: 4<br>Time: 5.0<br>Drain type: Default	False
4	Wash	Main solvent volume: 4<br>Time: 5.0<br>Drain type: Default	False
5	Wash	Main solvent volume: 4<br>Time: 5.0<br>Drain type: Default	False

0.05-Double Coupling (HS)

Step	Operation	Parameters	Pause
1	Deprotection	ID: Standard Deprotection<br>Deprotection volume: 3	False
2	Wash	Main solvent volume: 4<br>Time: 5<br>Drain type: Default	False
3	Wash	Main solvent volume: 4<br>Time: 5<br>Drain type: Default	False
4	Wash	Main solvent volume: 4<br>Time: 5<br>Drain type: Default	False
5	Wash	Main solvent volume: 4<br>Time: 5<br>Drain type: Default	False
6	Coupling	ID: Standard Coupling<br>Bottle position: (from method)<br>Bottle position volume: 1.25<br>Activator bottle position: PositionACT<br>Activator volume: 1<br>Activator base bottle position: PositionACTB<br>Activator base volume: 0.5<br>Delay reagent time: 0<br>Delay reagent bottle position: PositionACTB<br>Delay reagent volume: 0<br>Main solvent volume: 2<br>Value1: 1	False
7	Coupling	ID: Standard Coupling<br>Bottle position: (from method)<br>Bottle position volume: 1.25<br>Activator bottle position: PositionACT<br>Activator volume: 1<br>Activator base bottle position: PositionACTB<br>Activator base volume: 0.5<br>Delay reagent time: 0<br>Delay reagent bottle position: PositionACTB<br>Delay reagent volume: 0<br>Main solvent volume: 2<br>Value1: 1	False
8	Wash	Main solvent volume: 4<br>Time: 5.0<br>Drain type: Default	False

Figure S13. Standard cycles used for all syntheses. “Single coupling” cycles include one fewer coupling step than “double coupling” cycles.

Sequence: K C N T A T C 7 Q R L A N F L V H S S N N 2 G A I 5 S T N V G S N T Y - $CONH_2$

Resin	
Name	
Scale	0.05 mmol
Loading	0.19 mmol/g
Type	High-Swelling
Preloaded	No
C-Terminus	Amide
Mass Required	0.263 g

Cycles	
Resin	0.05-No Resin Swelling (HS)
Final Deprotection	0.05-Final Deprotection (HS)

Amino Acid	Specific Derivative	Cycle
Lys - K	Fmoc-Lys(Boc)-OH	0.05-Double Coupling (HS)
Cys - C	Fmoc-Cys(Trt)-OH	0.05-Double Coupling (HS)
Asn - N	Fmoc-Asn(Trt)-OH	0.05-Double Coupling (HS)
Thr - T	Fmoc-Thr(tBu)-OH	0.05-Double Coupling (HS)
Ala - A	Fmoc-Ala-OH	0.05-Double Coupling (HS)
Thr - T	Fmoc-Thr(tBu)-OH	0.05-Double Coupling (HS)
Cys - C	Fmoc-Cys(Trt)-OH	0.05-Double Coupling (HS)
EX7 - 7	External Amino Acid 7	0.05-Double 4min 90C Coupling (HS)
Gln - Q	Fmoc-Gln(Trt)-OH	0.05-Single 4min 90C Coupling (HS)
Arg - R	Fmoc-Arg(Pbf)-OH	0.05-Double Coupling (HS)
Leu - L	Fmoc-Leu-OH	0.05-Single 4min 90C Coupling (HS)
Ala - A	Fmoc-Ala-OH	0.05-Single 4min 90C Coupling (HS)
Asn - N	Fmoc-Asn(Trt)-OH	0.05-Single 4min 90C Coupling (HS)
Phe - F	Fmoc-Phe-OH	0.05-Single 4min 90C Coupling (HS)
Leu - L	Fmoc-Leu-OH	0.05-Single 4min 90C Coupling (HS)
Val - V	Fmoc-Val-OH	0.05-Single 4min 90C Coupling (HS)
His - H	Fmoc-His(Trt)-OH	0.05-Single 50°C 10 min Coupling (HS)
Ser - S	Fmoc-Ser(tBu)-OH	0.05-Single Coupling (HS)
Ser - S	Fmoc-Ser(tBu)-OH	0.05-Single Coupling (HS)
Asn - N	Fmoc-Asn(Trt)-OH	0.05-Single Coupling (HS)
Asn - N	Fmoc-Asn(Trt)-OH	0.05-Single Coupling (HS)
EX2 - 2	External Amino Acid 2	0.05-Single 10min 90C Coupling -xtra Washes (HS)
Gly - G	Fmoc-Gly-OH	0.05-Single Coupling (HS)
Ala - A	Fmoc-Ala-OH	0.05-Single Coupling (HS)
Ile - I	Fmoc-Ile-OH	0.05-Single Coupling (HS)
EX5 - 5	External Amino Acid 5	0.05-Single Coupling (HS)
Ser - S	Fmoc-Ser(tBu)-OH	0.05-Single Coupling (HS)
Thr - T	Fmoc-Thr(tBu)-OH	0.05-Single Coupling (HS)
Asn - N	Fmoc-Asn(Trt)-OH	0.05-Single Coupling (HS)
Val - V	Fmoc-Val-OH	0.05-Single Coupling (HS)
Gly - G	Fmoc-Gly-OH	0.05-Single Coupling (HS)
Ser - S	Fmoc-Ser(tBu)-OH	0.05-Single Coupling (HS)
Asn - N	Fmoc-Asn(Trt)-OH	0.05-Single Coupling (HS)
Thr - T	Fmoc-Thr(tBu)-OH	0.05-Single Coupling (HS)
Tyr - Y	Fmoc-Tyr(tBu)-OH	0.05-double deprotection, single coupling 4min 90C Coupling (HS)

Figure S14. Protocol used for the mono and di hIAPP analogues syntheses.

Sequence: K C N T A T C 7 Q R L A N F L V H S S N N 2 G A I 5 S T N V G S N T Y - $CONH_2$

Resin	
Name	
Scale	0.05 mmol
Loading	0.19 mmol/g
Type	High-Swelling
Preloaded	No
C-Terminus	Amide
Mass Required	0.263 g

Cycles	
Resin	0.05-No Resin Swelling (HS)
Final Deprotection	0.05-Final Deprotection (HS)

Amino Acid	Specific Derivative	Cycle
Lys - K	Fmoc-Lys(Boc)-OH	0.05-Double Coupling (HS)
Cys - C	Fmoc-Cys(Trt)-OH	0.05-Double Coupling (HS)
Asn - N	Fmoc-Asn(Trt)-OH	0.05-Double Coupling (HS)
Thr - T	Fmoc-Thr(tBu)-OH	0.05-Double Coupling (HS)
Ala - A	Fmoc-Ala-OH	0.05-Double Coupling (HS)
Thr - T	Fmoc-Thr(tBu)-OH	0.05-Double Coupling (HS)
Cys - C	Fmoc-Cys(Trt)-OH	0.05-Double Coupling (HS)
EX7 - 7	External Amino Acid 7	0.05-Double 4min 90C Coupling (HS)
Gln - Q	Fmoc-Gln(Trt)-OH	0.05-Single 4min 90C Coupling (HS)
Arg - R	Fmoc-Arg(Pbf)-OH	0.05-Double Coupling (HS)
Leu - L	Fmoc-Leu-OH	0.05-Single 4min 90C Coupling (HS)
Ala - A	Fmoc-Ala-OH	0.05-Single 4min 90C Coupling (HS)
Asn - N	Fmoc-Asn(Trt)-OH	0.05-Single 4min 90C Coupling (HS)
Phe - F	Fmoc-Phe-OH	0.05-Single 4min 90C Coupling (HS)
Leu - L	Fmoc-Leu-OH	0.05-Single 4min 90C Coupling (HS)
Val - V	Fmoc-Val-OH	0.05-Single 4min 90C Coupling (HS)
His - H	Fmoc-His(Trt)-OH	0.05-Single 50°C 10 min Coupling (HS)
Ser - S	Fmoc-Ser(tBu)-OH	0.05-Single 4min 90C Coupling (HS)
Ser - S	Fmoc-Ser(tBu)-OH	0.05-Single 4min 90C Coupling (HS)
Asn - N	Fmoc-Asn(Trt)-OH	0.05-Single 4min 90C Coupling (HS)
Asn - N	Fmoc-Asn(Trt)-OH	0.05-Double 4min 90C Coupling (HS)
EX2 - 2	External Amino Acid 2	0.05-Single 10min 90C Coupling -xtra Washes (HS)
Gly - G	Fmoc-Gly-OH	0.05-Single Coupling (HS)
Ala - A	Fmoc-Ala-OH	0.05-Single Coupling (HS)
Ile - I	Fmoc-Ile-OH	0.05-Single Coupling (HS)
EX5 - 5	External Amino Acid 5	0.05-Single Coupling (HS)
Ser - S	Fmoc-Ser(tBu)-OH	0.05-Single Coupling (HS)
Thr - T	Fmoc-Thr(tBu)-OH	0.05-Single Coupling (HS)
Asn - N	Fmoc-Asn(Trt)-OH	0.05-Single Coupling (HS)
Val - V	Fmoc-Val-OH	0.05-Single Coupling (HS)
Gly - G	Fmoc-Gly-OH	0.05-Single Coupling (HS)
Ser - S	Fmoc-Ser(tBu)-OH	0.05-Single Coupling (HS)
Asn - N	Fmoc-Asn(Trt)-OH	0.05-Single Coupling (HS)
Thr - T	Fmoc-Thr(tBu)-OH	0.05-Single Coupling (HS)
Tyr - Y	Fmoc-Tyr(tBu)-OH	0.05-double deprotection, single coupling 4min 90C Coupling (HS)

Figure S15. Protocol used for the tetra and penta hIAPP analogues syntheses.

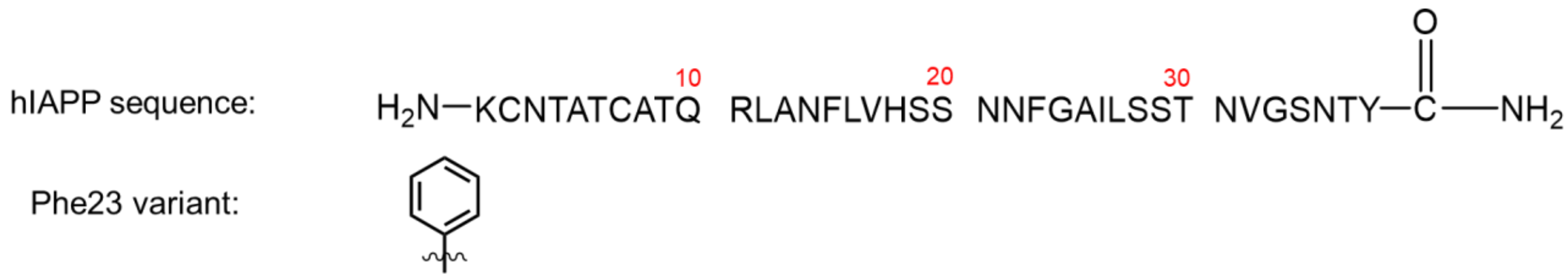

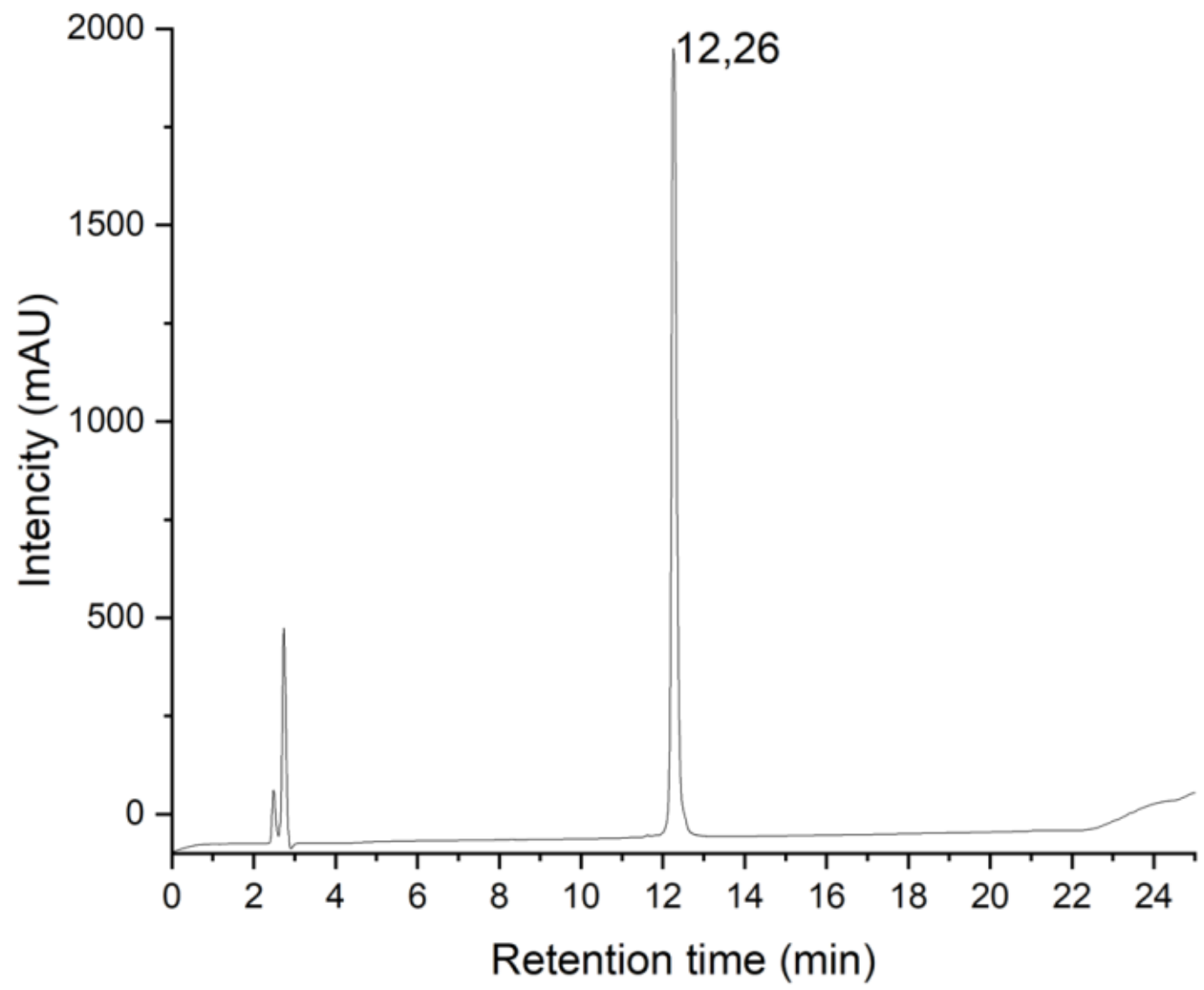

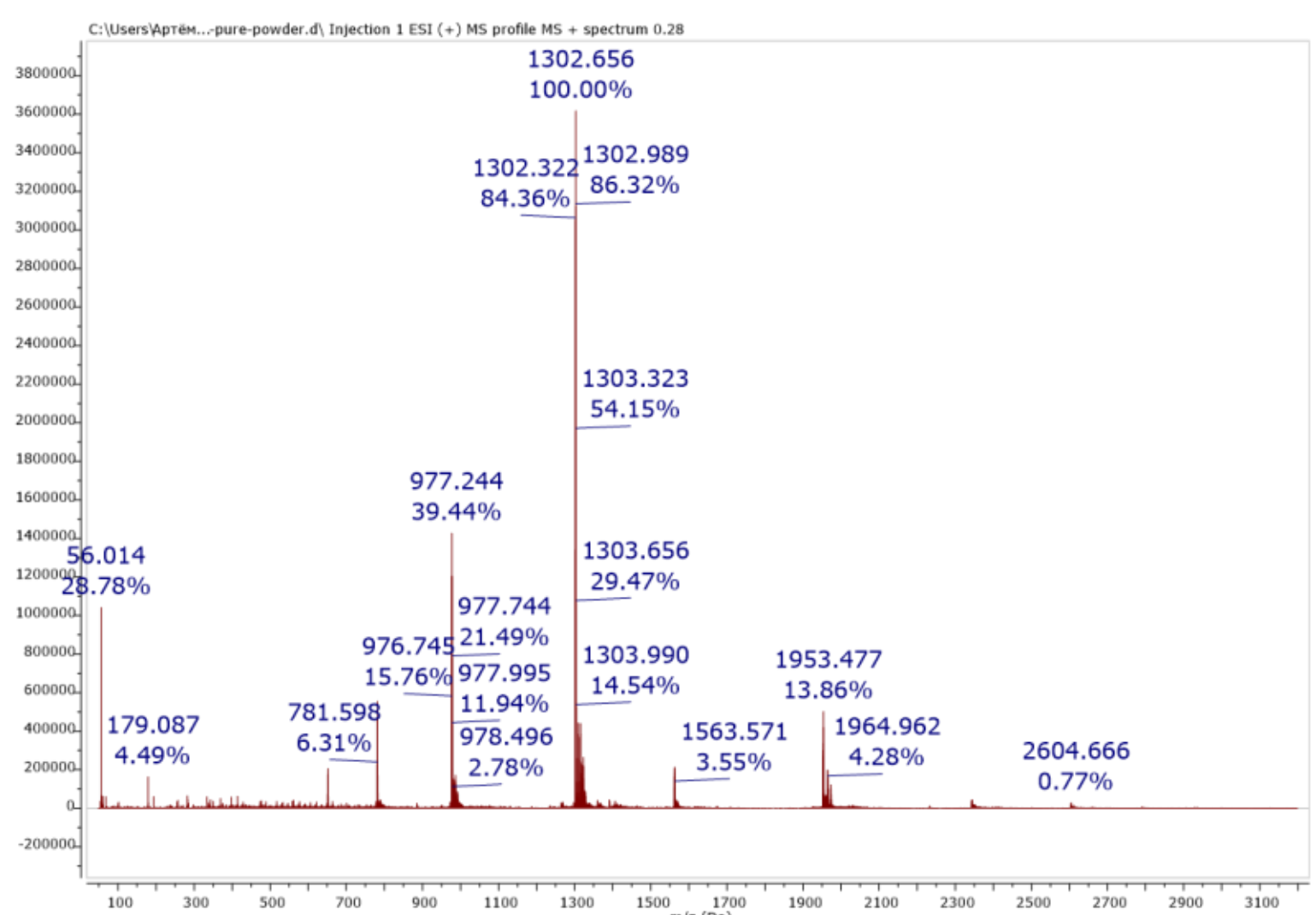

monoisotopic mass calculation for peptide:
N-terminal group : free amine
C-terminal group : amide

Expected Molecular mass :
3902.8791

Electrospray series (positive mode) :

charge	m/z
+1	3903.8869
+2	1952.4474
+3	1301.9675
+4	976.7276
+5	781.5836

Figure S16. Analytical HPLC chromatogram and MS spectrum of wt hIAPP.

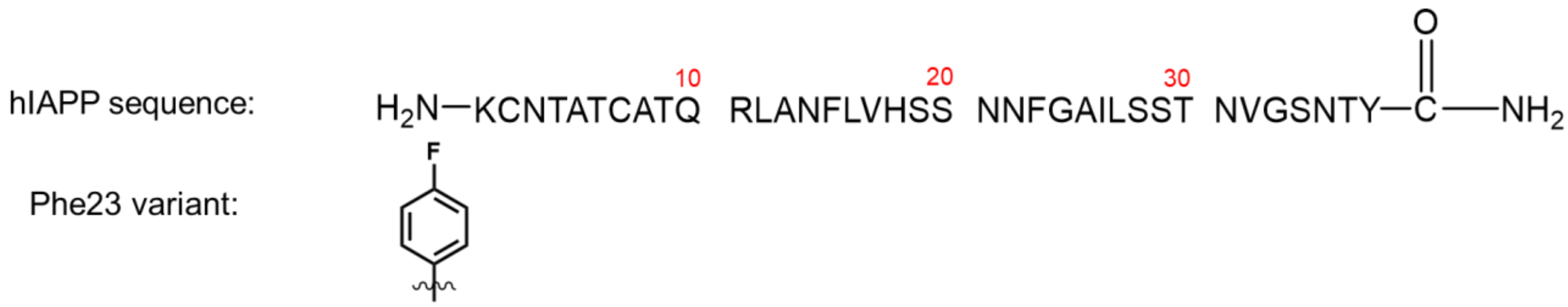

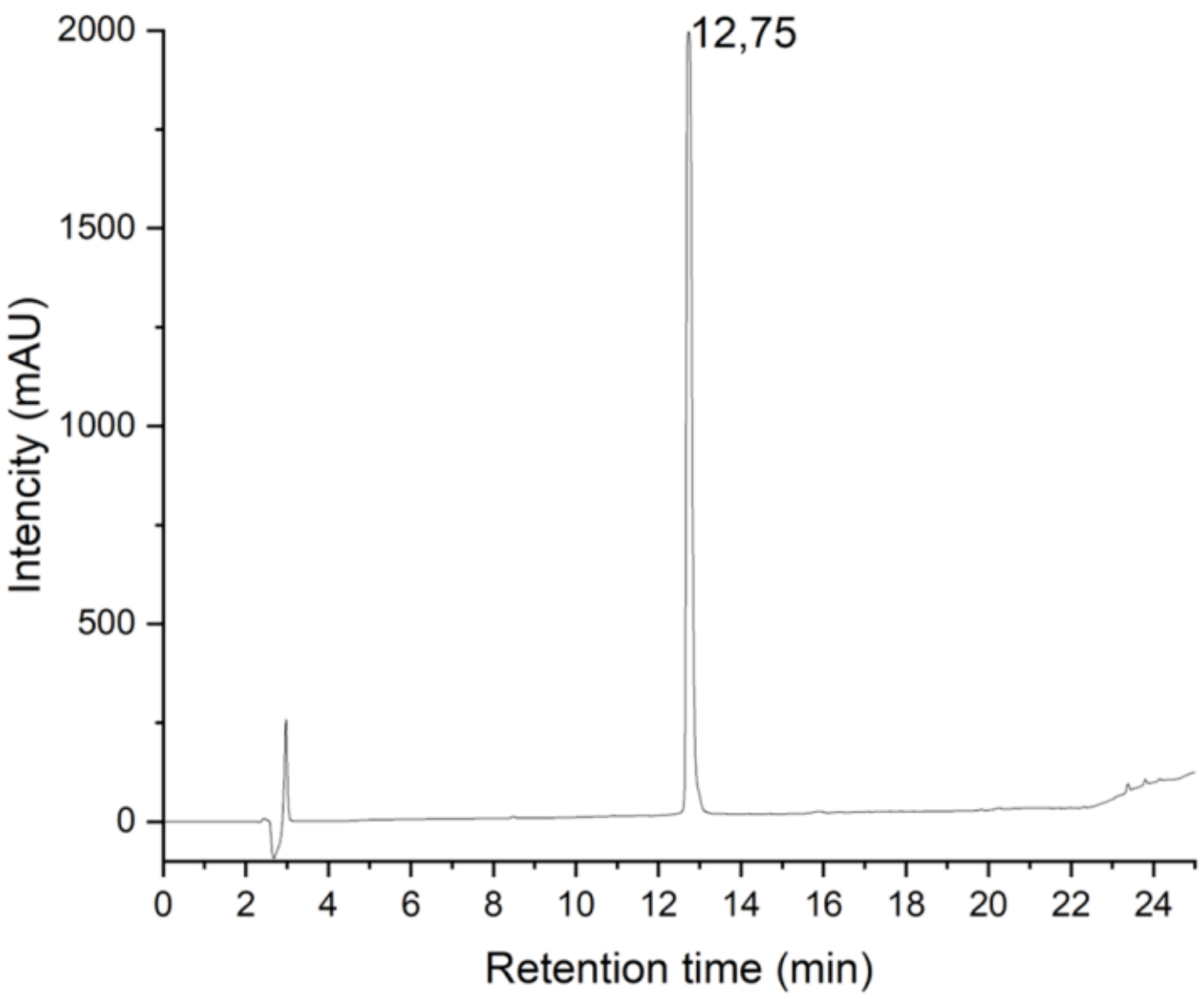

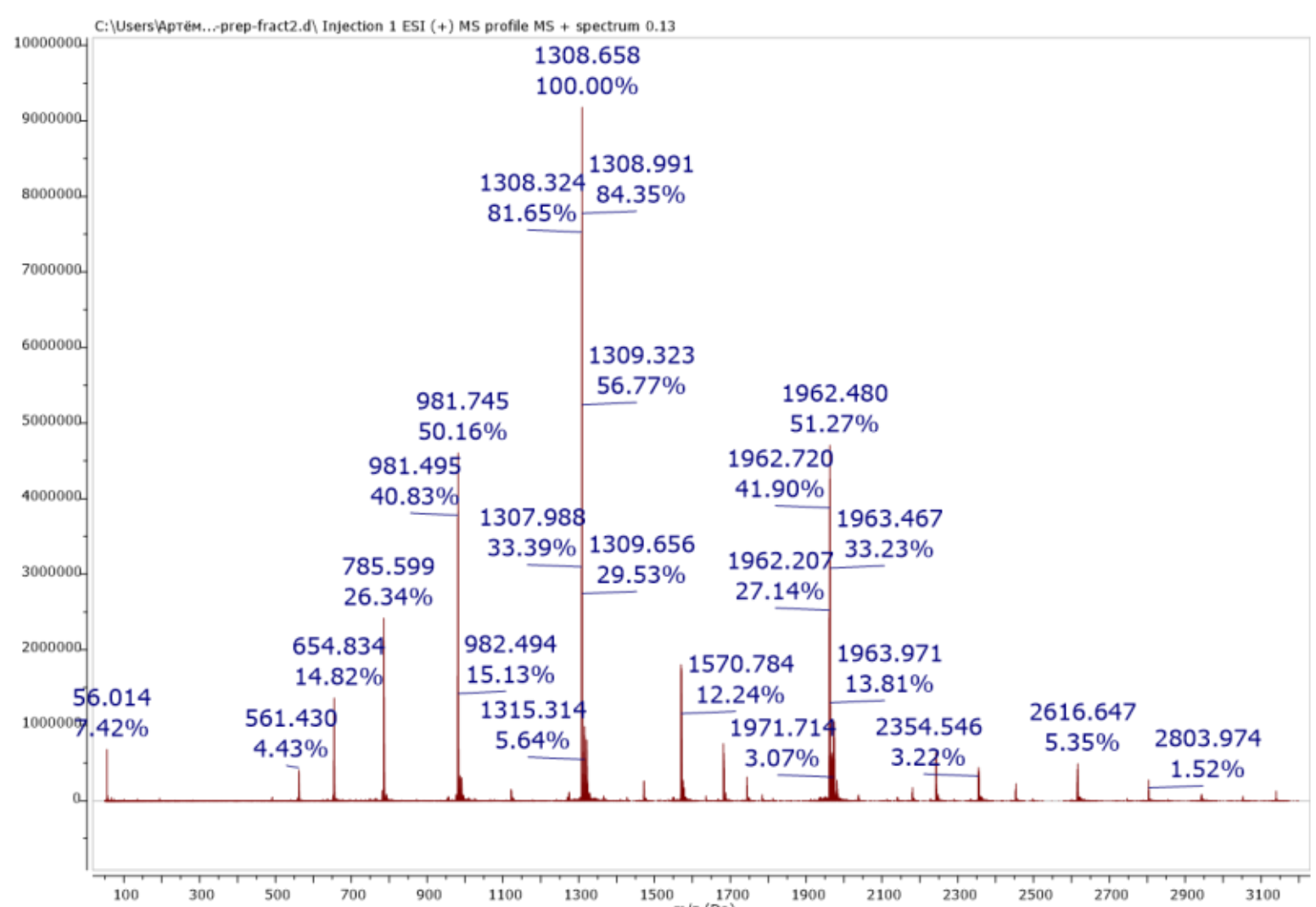

monoisotopic mass calculation for peptide:
N-terminal group : free amine
C-terminal group : amide

Expected Molecular mass :
3922

Electrospray series (positive mode) :

charge	m/z
+1	3923
+2	1962
+3	1308
+4	982
+5	785

Figure S17. Analytical HPLC chromatogram and MS spectrum of mono hIAPP variant.

hIAPP sequence:

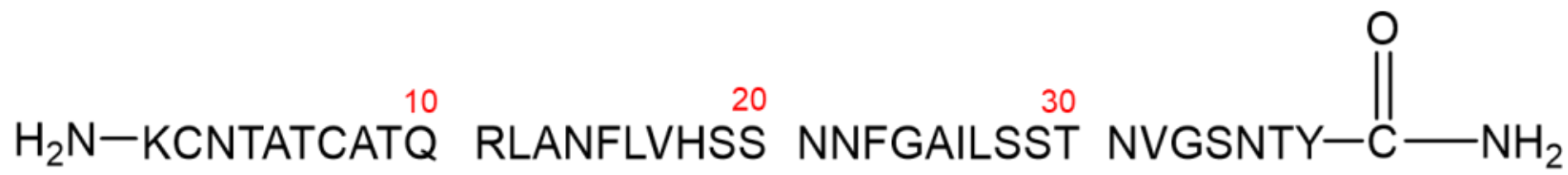

Phe23 variant:

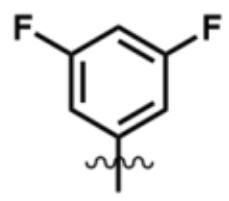

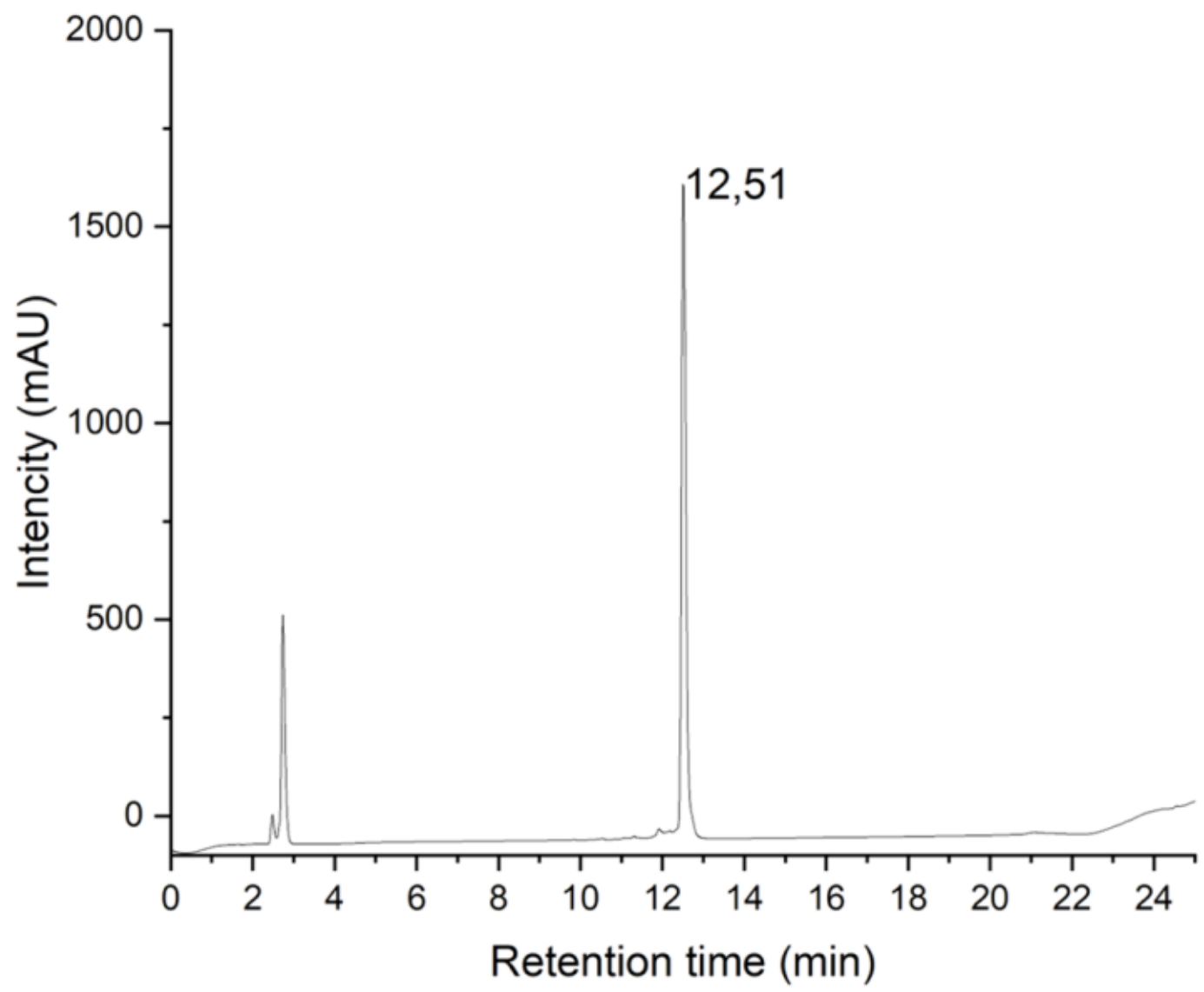

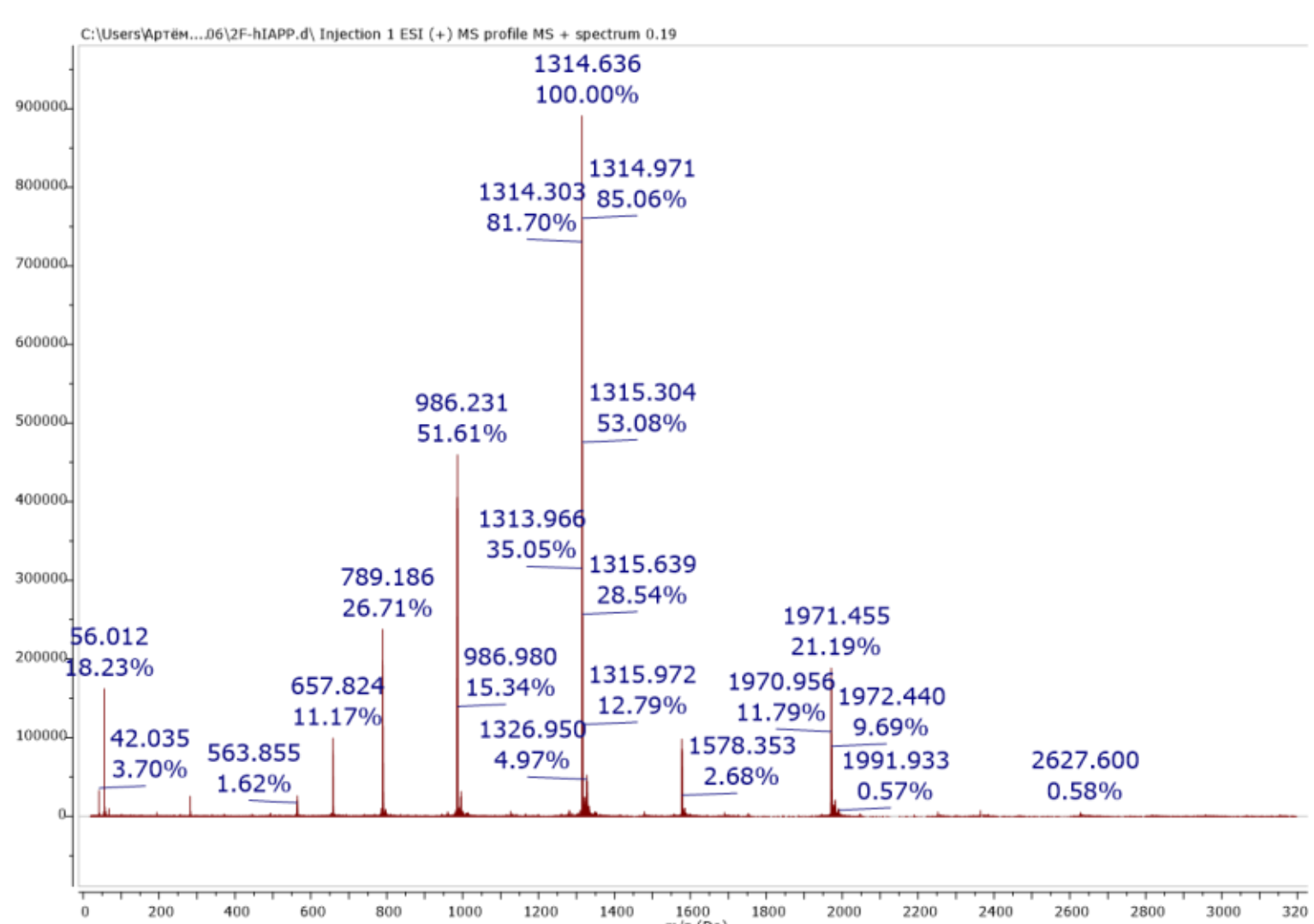

monoisotopic mass calculation for peptide:
N-terminal group : free amine
C-terminal group : amide

Expected Molecular mass :
3941

Electrospray series (positive mode) :

charge	m/z
+1	3942
+2	1972
+3	1315
+4	986
+5	789

Figure S18. Analytical HPLC chromatogram and MS spectrum of di hIAPP variant.

hIAPP sequence: H_2N—KCNTATCATQ[10] RLANFLVHSS[20] NNFGAILSST[30] NVGSNTY—C(=O)—NH_2

Phe23 variant:

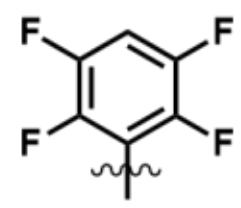

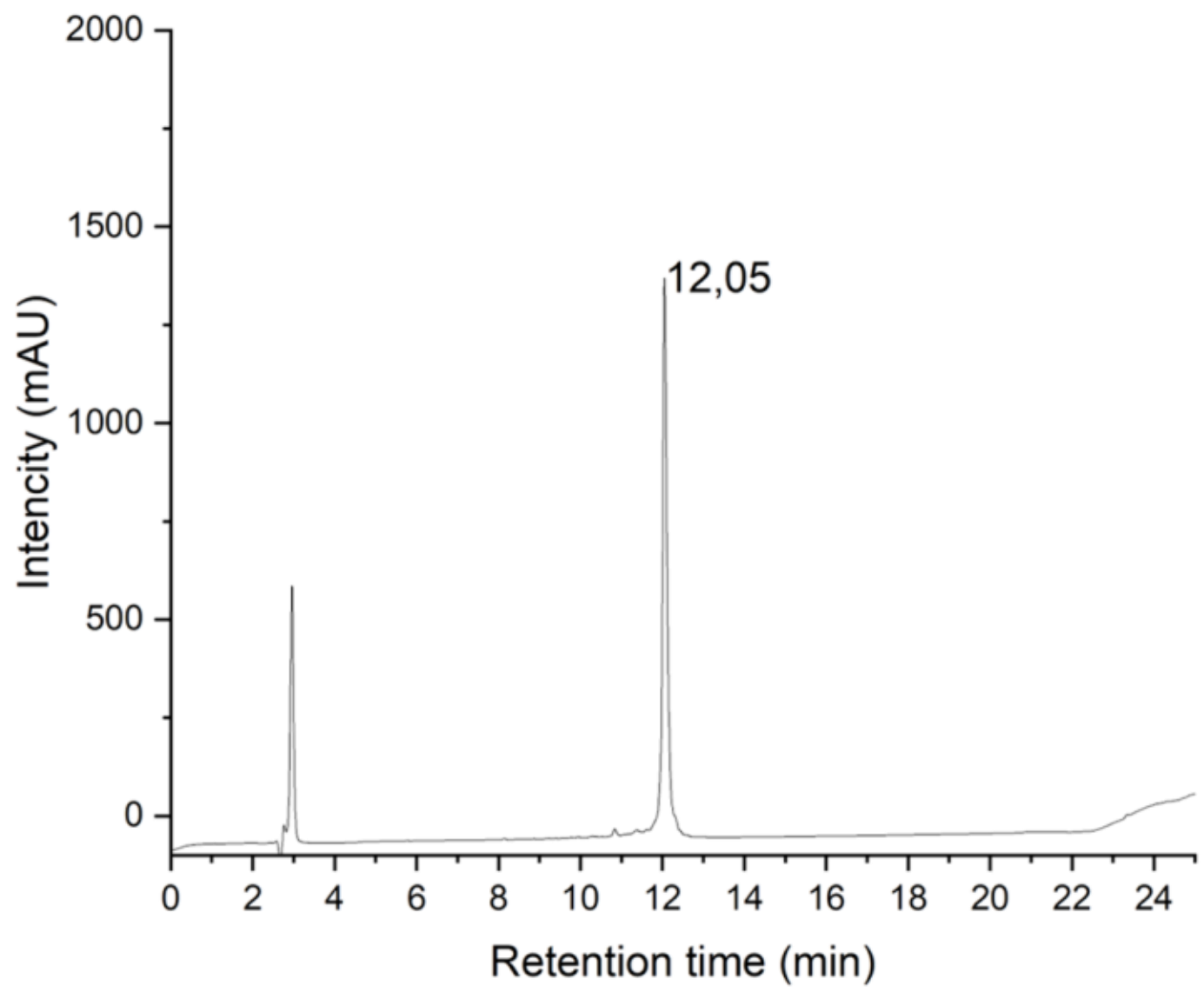

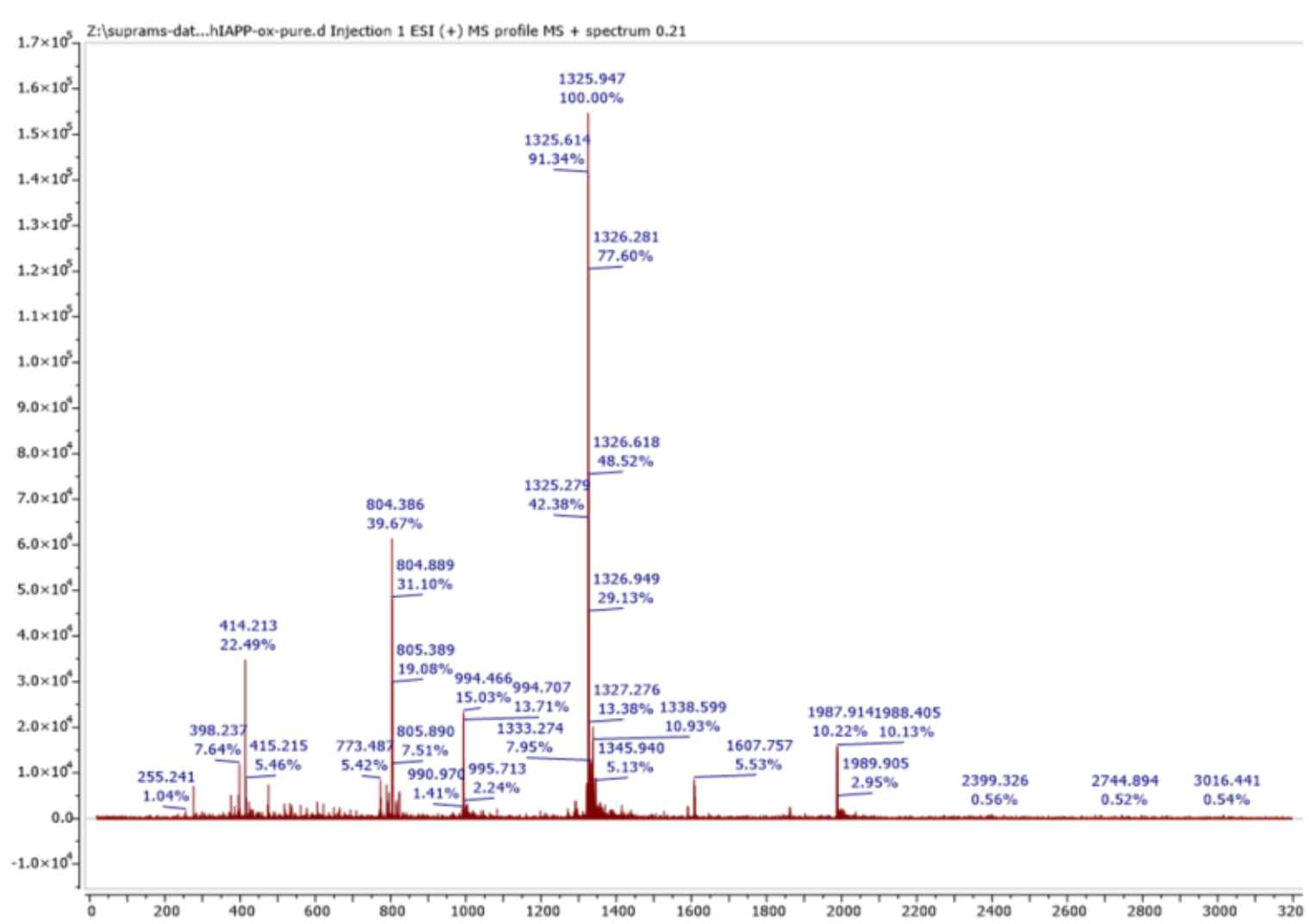

monoisotopic mass calculation for peptide:
N-terminal group : free amine
C-terminal group : amide

Expected Molecular mass :
3979

Electrospray series (positive mode) :

charge	m/z
+1	3980
+2	1991
+3	1327
+4	996
+5	797

Figure S19. Analytical HPLC chromatogram and MS spectrum of tetra hIAPP variant

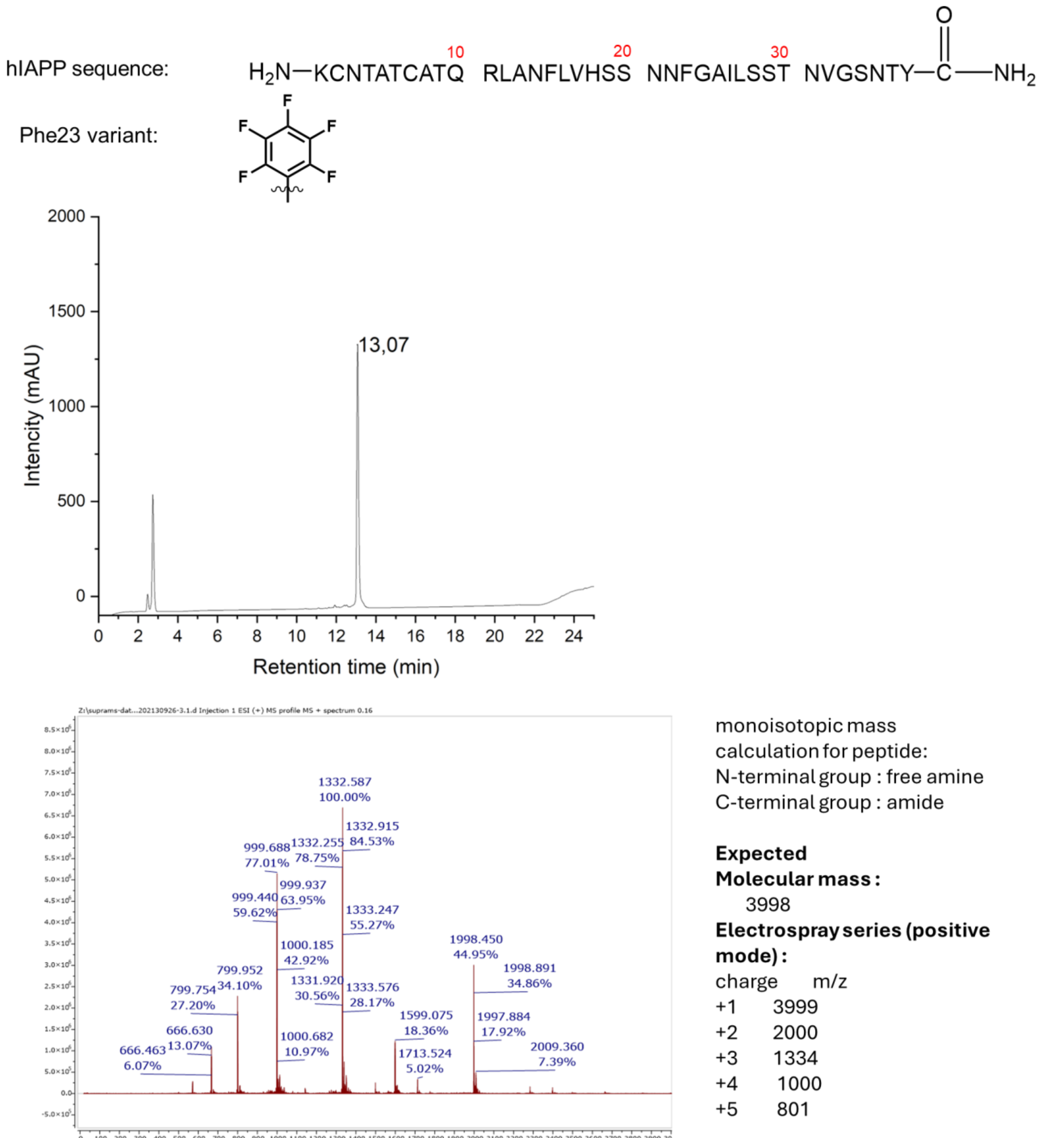

Figure S20. Analytical HPLC chromatogram and MS spectrum of penta hIAPP variant
